# Responses of denitrifying bacterial communities to short-term waterlogging of soils

**DOI:** 10.1038/s41598-017-00953-8

**Published:** 2017-04-11

**Authors:** Yong Wang, Yoshitaka Uchida, Yumi Shimomura, Hiroko Akiyama, Masahito Hayatsu

**Affiliations:** 1 Institute for Agro-Environmental Sciences, National Agriculture and Food Research Organization (NARO), 3-1-3, Kannondai, Tsukuba, Ibaraki 305-8604 Japan; 2 Research Faculty of Agriculture, Hokkaido University, Kita 9, Nishi 9, Kita-ku, Sapporo, Hokkaido 060-8589 Japan; 3 Kyodo Milk Industry Co., Ltd, 20-1, Hirai, Hinode, Nishitama, Tokyo 190-0182 Japan

## Abstract

Agricultural soil is often subjected to waterlogging after heavy rainfalls, resulting in sharp and explosive increases in the emission of nitrous oxide (N_2_O), an important greenhouse gas primarily released from agricultural soil ecosystems. Previous studies on waterlogged soil examined the abundance of denitrifiers but not the composition of denitrifier communities in soil. Also, the PCR primers used in those studies could only detect partial groups of denitrifiers. Here, we performed pyrosequencing analyses with the aid of recently developed PCR primers exhibiting high coverage for three denitrification genes, *nirK*, *nirS*, and *nosZ* to examine the effect of short-term waterlogging on denitrifier communities in soil. We found that microbial communities harboring denitrification genes in the top 5 cm of soil distributed according to soil depth, water-soluble carbon, and nitrate nitrogen. Short-term waterlogging scarcely affected abundance, richness, or the alpha-diversities of microbial communities harboring *nirK*, *nirS*, and *nosZ* genes, but significantly affected their composition, particularly in microbial communities at soil depths of 0 to 1 cm. Our results indicated that the composition of denitrifying microbial communities but not the abundance of denitrifiers in soil was responsive to short-term waterlogging of an agricultural soil ecosystem.

## Introduction

Nitrous oxide (N_2_O) is a greenhouse gas that is mainly released from soil ecosystems^1^. Thus it has received considerable attention from soil scientists in recent decades^2–4^. Agricultural soil produces N_2_O during nitrification and denitrification processes^5, 6^. During denitrification, N_2_O emission from soil is affected by oxygen levels, soil moisture, and substrate availability^7^. The oxygen level in soil is often the most critical factor, because true denitrification occurs under anaerobic conditions^7^. Moisture affects denitrification by altering oxygen supply to the soil^8^. For example, large N_2_O emissions are observed immediately following irrigation or rainfall^9–16^. Specifically, Akiyama *et al*. observed large N_2_O emissions (up to 1.59 kg N ha^−1^ day^−1^) following heavy rainfalls in an upland converted paddy field, accounting for 55% to 80% of the annual N_2_O emission from that field^15^. Additionally, we observed large N_2_O emissions using temporarily (24 h) waterlogged intact soil cores sampled from the same field^17^. Knowledge of the composition of corresponding microbial communities is required to link rapid increases in N_2_O emissions with environmental changes^18^. Therefore, to understand the mechanisms underlying N_2_O emission following short-term waterlogging by rainfall, it is necessary to investigate the community composition of denitrifying microbes in soils and their responses to short-term waterlogging.

Molecular tools can be used to elucidate relationships between soil microbes and N_2_O emissions^19^. The information obtained from soil microbial DNA reveals the community composition and abundance of denitrifiers contributing to N_2_O emission in soil^18, 20^. To understand the composition of and variations in denitrifier communities in soil, pyrosequencing of PCR amplicons from denitrification genes generated from soil nucleic acids can be used. Similar to other PCR-based detection techniques, PCR primers are key factors that determine the reliability of pyrosequencing results. Recently, substantial efforts have been devoted to developing primers for denitrification genes in order to detect more microbial taxa^21–30^. Among these primers, those recently developed for *nirK* (encoding a copper-containing nitrite reductase)^28^, *nirS* (encoding a cytochrome cd_1_-containing nitrite reductase)^28^, and *nosZ* (encoding a nitrous oxide reductase)^29, 31^ exhibit significantly higher coverage of microbial taxa compared with others. Therefore, in this study, we used conventional PCR, quantitative PCR (qPCR), and pyrosequencing techniques, as well as recently developed PCR primers, to investigate communities of microbes harboring *nirK*, *nirS*, and *nosZ* genes in soil from an upland converted paddy field that exhibited active N_2_O emission immediately following heavy rainfalls.

## Results

### Detection of denitrification genes in soil cores

Existence of denitrification genes in raw soil samples was examined using conventional PCR and primers targeting the *nirK* gene in clusters I, II, III, and IV, the *nirS* gene in clusters I, II, and III, and the *nosZ* gene in clades I and II. Our results indicated detection of *nirK* in cluster II, *nirS* in cluster I, and *nosZ* in clades I and II (see Supplementary Fig. S1), suggesting that these sequences were dominant in these soil samples. Therefore, in this study, we used these four sets of PCR primers to analyze microbial communities harboring these denitrification genes in soil samples.

**Figure 1:**
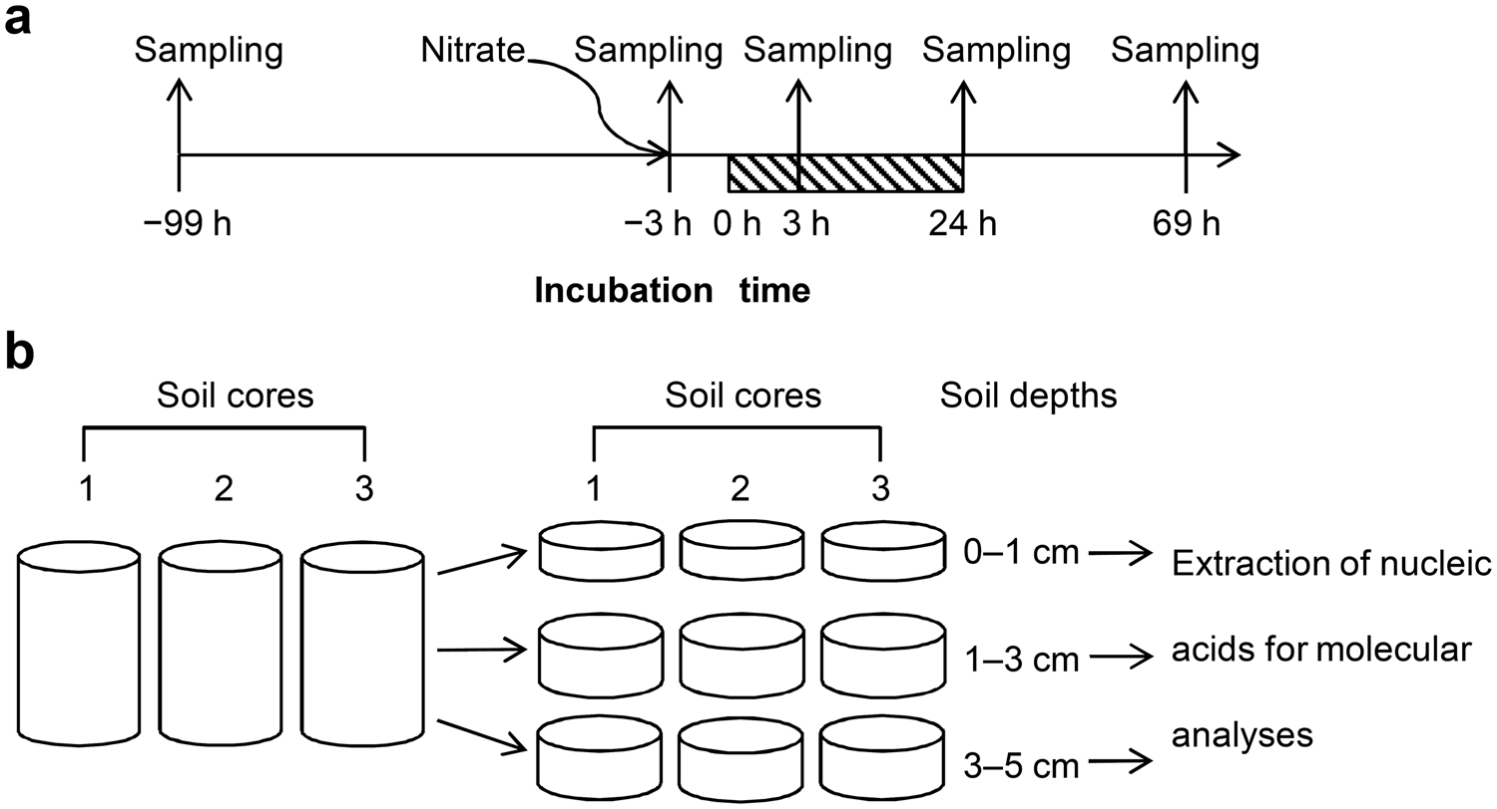
Sampling time (**a**) and dissection (**b**) of soil cores. The shadowed region indicates the waterlogging period. Arrows indicate the time of soil sampling and addition of nitrate.

**Figure 2:**
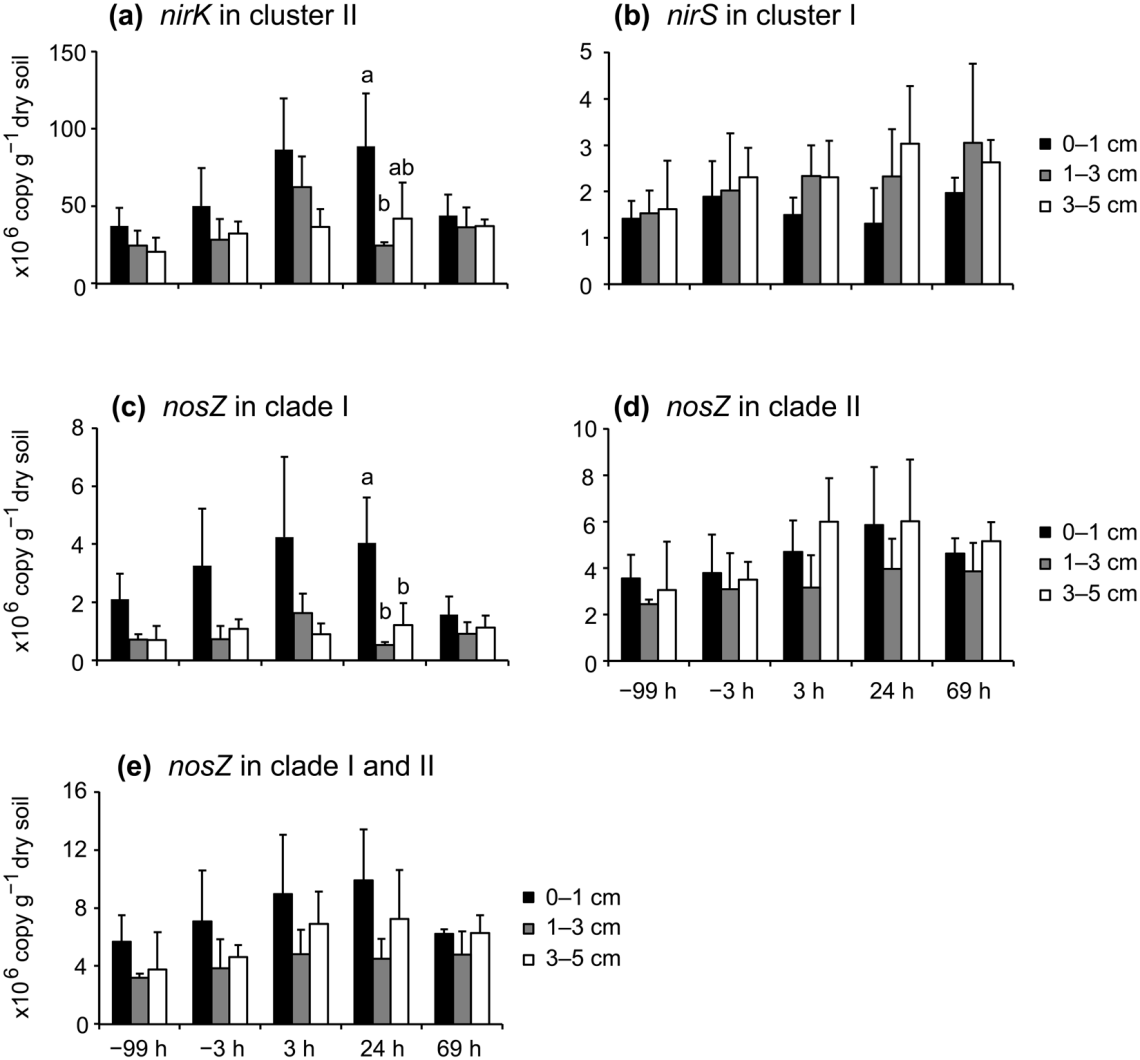
Quantification of the abundance of *nirK* in cluster II (**a**), *nirS* in cluster I (**b**), *nosZ* in clade I (**c**), *nosZ* in clade II (**d**), and the total quantity of *nosZ* (*nosZ* in clades I and II) (**e**) in soil by qPCR. Error bars represent standard deviations (*n* = 3). Different lowercase letters above columns represent significant differences among different soil depths at each sampling time.

qPCR was performed to examine the abundance of denitrification genes in the soil samples (Fig. 1). As shown in Fig. 2, no gene showed significant alterations in copy number, suggesting no significant effect of waterlogging on the abundance of microbes harboring these genes. The copy number of *nirK* in cluster II was ~10- to ~70-fold higher than that of *nirS* in cluster I (Fig. 2a and b), and because the enzymes encoded by the *nirK* and *nirS* genes catalyze the same chemical reaction (reduction of nitrite to nitric oxide) during the denitrification process, these data suggested that *nirK*, but not *nirS*, played a key role in denitrification in these soil samples. The copy number of *nosZ* in clade II, which is a newly described cluster of *nosZ* sequences, was ~1- to ~7-fold higher than that of *nosZ* in clade I (Fig. 2c and d), suggesting that the microbes harboring *nosZ* in clade II may contribute to denitrification to a greater degree than those harboring *nosZ* in clade I. These results were consistent with those of a recent metagenomic analysis of agricultural soils^32^.

### The alpha-diversities and community compositions of denitrifiers in soil

Across all soil samples, 42,223 (*nirK* in cluster II), 117,270 (*nirS* in cluster I), 146,651 (*nosZ* in clade I), and 44,173 (*nosZ* in clade II) sequences with average sequence lengths of 412 ± 4 base pairs (*nirK* in cluster II), 367 ± 3 base pairs (*nirS* in cluster I), 413 ± 9 base pairs (*nosZ* in clade I), and 287 ± 15 base pairs (*nosZ* in clade II) were obtained. The Good’s library coverages were >96% (*nirK* in cluster II), >99% (*nirS* in cluster I and *nosZ* in clade I), and >94% (*nosZ* in clade II) (see Supplementary Table S6), suggesting that the numbers of obtained sequences were sufficient for diversity analysis. Rarefaction curves are shown in Supplementary Fig. S2.

**Figure 3:**
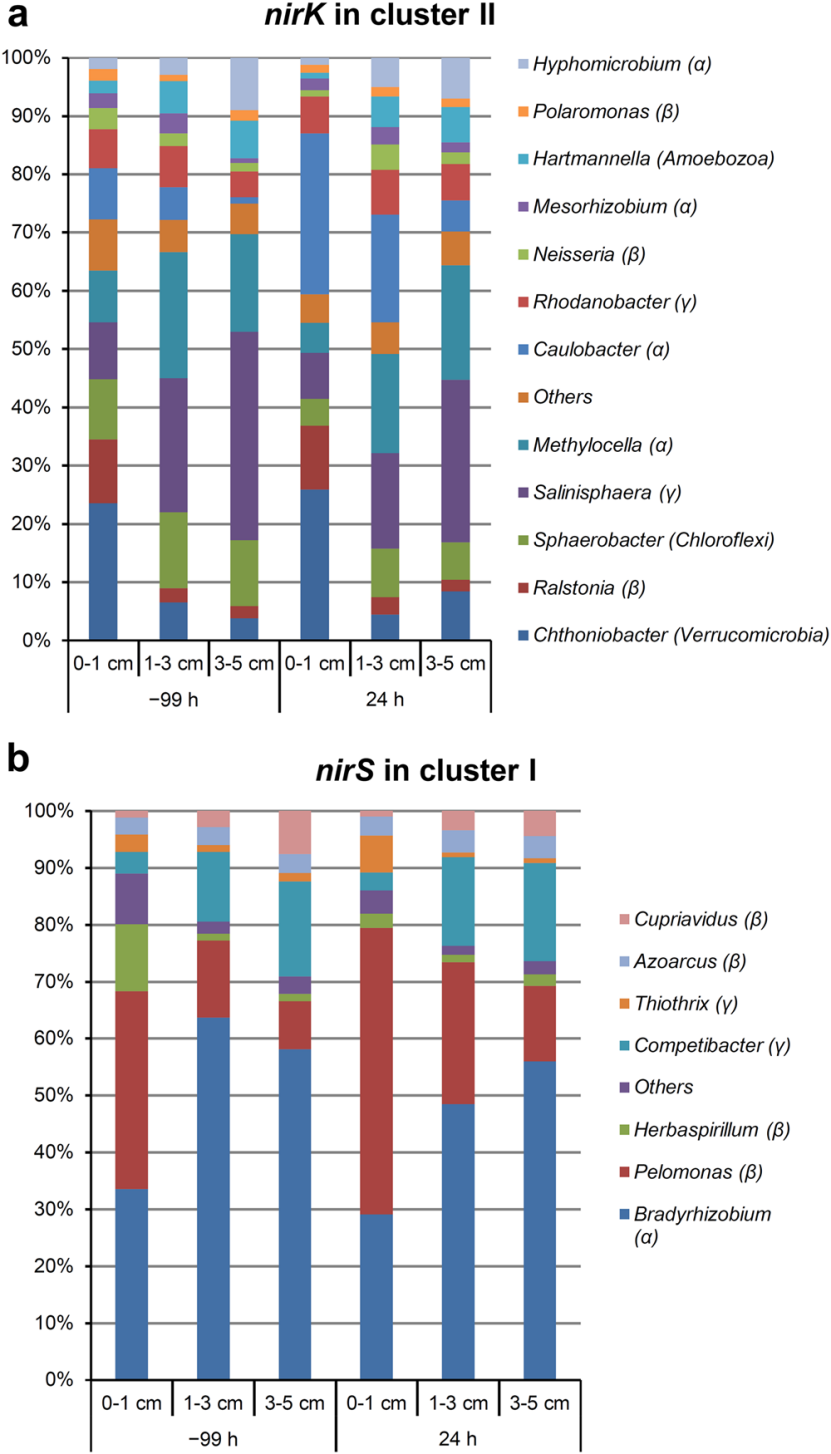
Average relative abundances of *nirK* in cluster II (**a**) and *nirS* in cluster I (**b**) sequences affiliated with taxonomic groups at the genus level. Each group is labeled with its genus name, with its phylum or subphylum name in parenthesis. Groups with <1% sequence number in the 18 soil samples were merged into the “Others” group. α, β, and γ represent *alpha-*, *beta-*, and *gamma-proteobacteria*, respectively.

**Figure 4:**
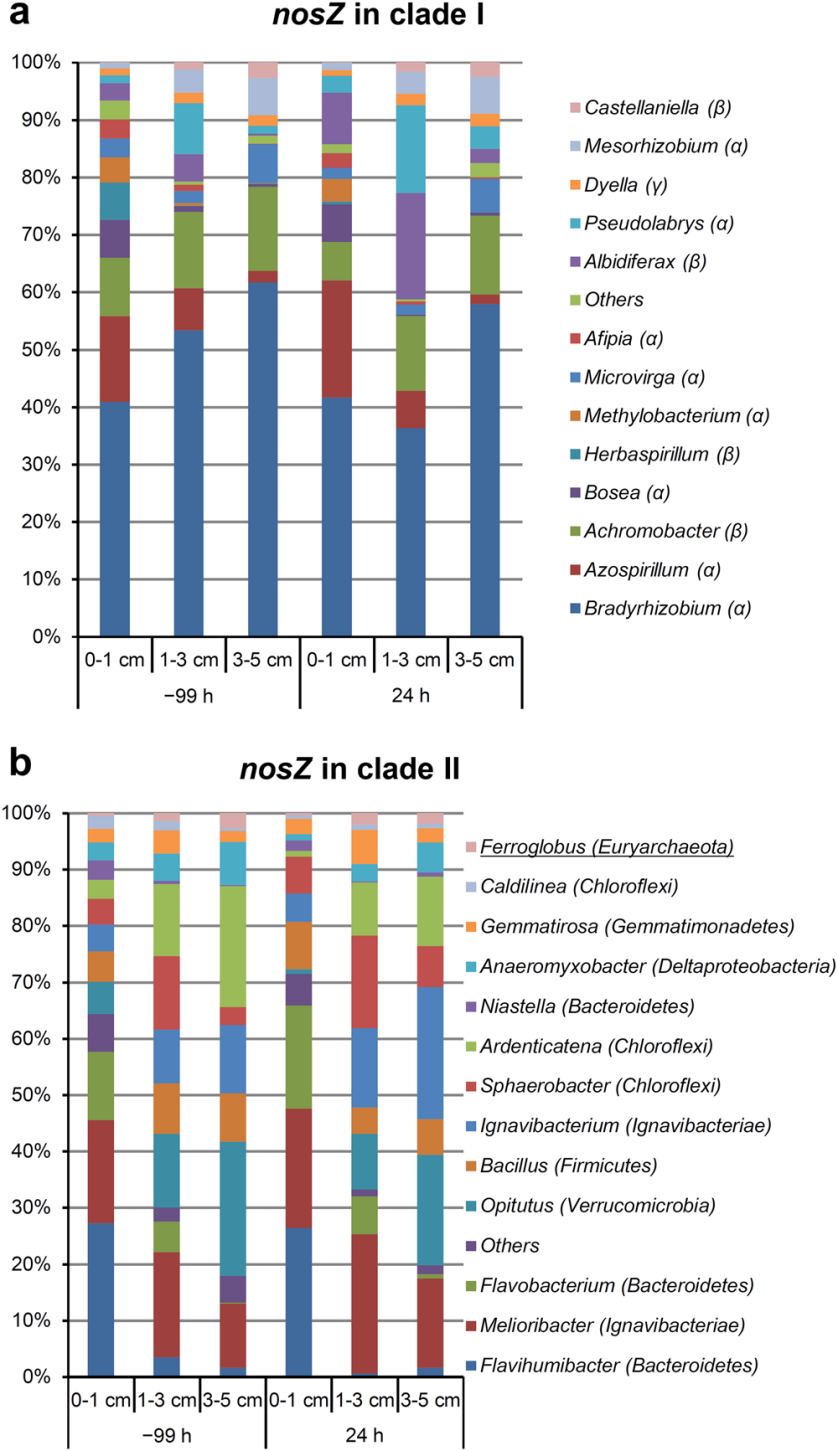
Average relative abundances of *nosZ* in clade I (**a**) and *nosZ* in clade II (**b**) sequences affiliated with taxonomic groups at the genus level. Each group is labeled with its genus name, with its phylum or subphylum name in parenthesis. Groups with <1% sequence number in the 18 soil samples were merged into the “Others” group. α, β, and γ represent *alpha-*, *beta-*, and *gamma-proteobacteria*, respectively. The underlined taxonomic name in (**b**) indicates an *Archaea* group.

The operational taxonomic unit (OTU) richness of all samples did not differ significantly according to one-way analysis of variance (ANOVA). Similar results were observed for the alpha-diversity indices, except for those of *nirK* in cluster II (see Supplementary Tables S6 and S7). Nearly 96% of *nirK* sequences in cluster II were affiliated with bacterial taxa, whereas >4% were affiliated with eukaryotes. The dominant taxa (the read number in a total of 18 soil samples was >1%) included *alpha-*, *beta*-, and *gamma*-*proteobacteria*, *Chloroflexi*, *Verrucomicrobia*, and *Amoebozoa* (Fig. 3a). With the exception of 32 sequences from fungi (*Fusarium*), all of the eukaryotic sequences were affiliated with genus *Hartmannella* of *Amoebozoa* (Fig. 3a). All of the dominant taxa for *nirS* in cluster I and *nosZ* in clade I were classified as *alpha*-, *beta*-, and *gamma*-*proteobacteria* (Figs 3b and 4a), suggesting low diversity at the phylum level. The dominant taxa for *nosZ* in clade II were classified into diverse phyla (Fig. 4b) belonging to two different kingdoms, *Archaea* and *Bacteria*, despite the archaeal sequences existing in <2% of 18 soil samples.

### Environmental factors shaping the alpha- and beta-diversities of microbes in soil

To characterize microbial communities in the soil prior to waterlogging (−99 h; Fig. 1), we examined correlations between the alpha-diversities of microbes in the −99-h soil cores and five environmental factors, including vertical distance (soil depth), horizontal distance between samples (soil core), water-soluble carbon, soil nitrogen in the form of nitrate (nitrate-N), and soil pH (see Supplementary Table S8). Spearman’s rank correlation test indicated that alpha-diversity indices of microbes harboring *nirK* in cluster II, but not other genes, positively correlated with water-soluble carbon, but negatively correlated with soil depth (see Supplementary Table S9), suggesting a decreased alpha-diversity of microbes harboring *nirK* in cluster II, with the increase in soil depth determined by water-soluble carbon.

**Figure 5:**
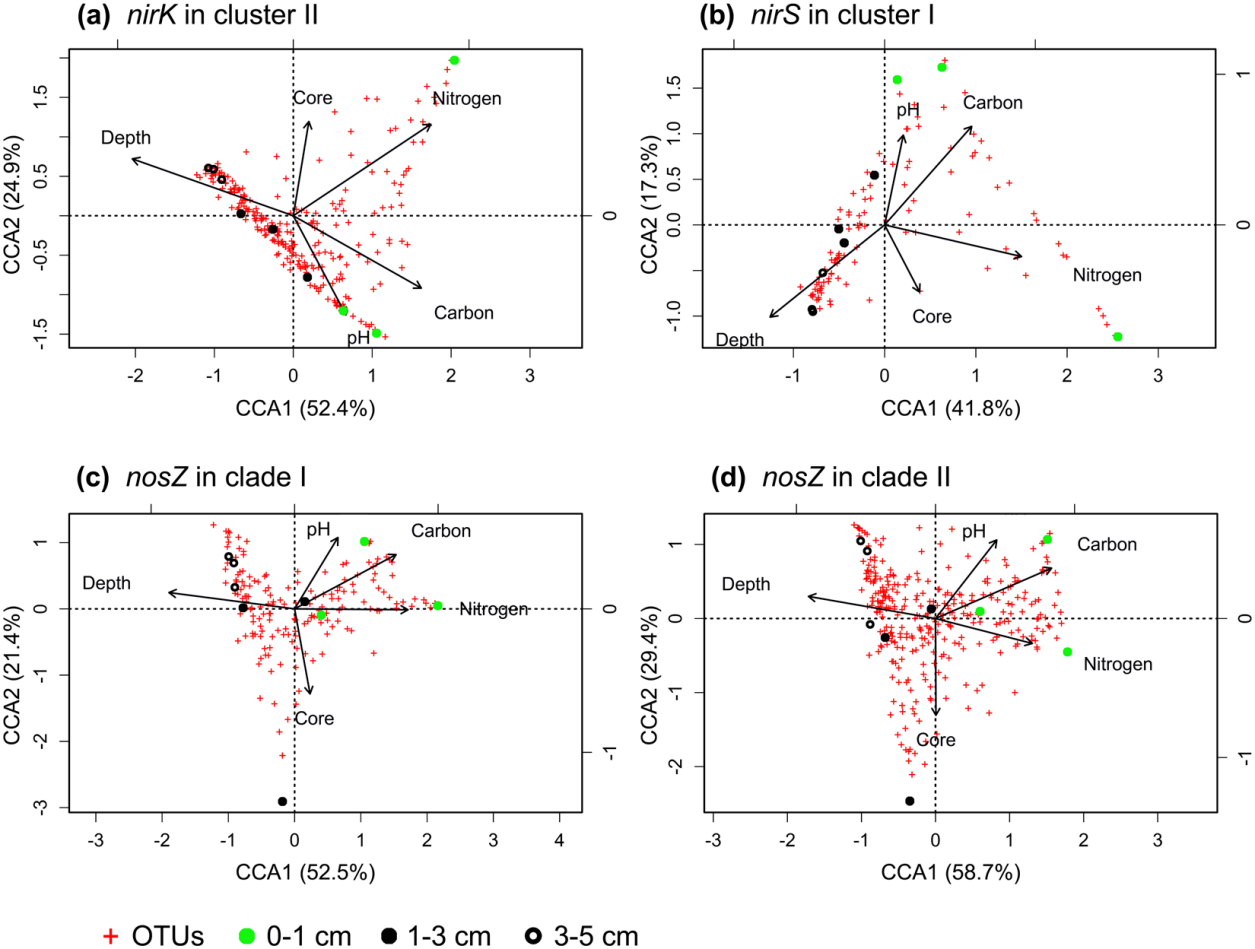
Evaluation of the effect of environmental factors on microbial communities harboring *nirK* in cluster II (**a**), *nirS* in cluster I (**b**), *nosZ* in clade I (**c**), and *nosZ* in clade II (**d**) in −99-h soil cores by constrained correspondence analysis.

We then performed constrained (or canonical) correspondence analysis (CCA) to determine the effect of environmental factors on the beta-diversities of denitrifiers harboring *nirK* in cluster II, *nirS* in cluster I, and *nosZ* in clades I and II in the soil samples (Fig. 5). The first two axes of the CCA explained 59% to 88% of the variations in these denitrification genes. Permutation tests on CCA results indicated that the effect of environmental factors on the beta-diversities of the denitrifiers was significant (number of permutations = 1000 in all cases; *nirK* in cluster II, F = 3.03, *p* = 0.001; *nirS* in cluster I, F = 6.82, *p* = 0.001; *nosZ* in clade I, F = 3.43, *p* = 0.001; *nosZ* in clade II, F = 2.62, *p* = 0.001). Consistent with the CCA data, permutational multivariate ANOVA (PERMANOVA; two-way ANOVA) results suggested that soil depth, water-soluble carbon, and nitrate-N significantly affected the beta-diversities of microbes harboring these genes (see Supplementary Table S10). According to partitioning analysis, the combined effect of soil depth, water-soluble carbon, and nitrate-N explained 76% (*nirK* in cluster II), 65% (*nirS* in cluster I), 43% (*nosZ* in clade I), and 51% (*nosZ* in clade II) of the observed variations (see Supplementary Fig. S3), suggesting that these environmental factors were important for shaping the beta-diversities of microbes harboring these genes in soil. Soil depth was particularly important, because its effect explained 63% (*nirK* in cluster II), 44% (*nirS* in cluster I), 34% (*nosZ* in clade I), and 35% (*nosZ* in clade II) of the observed variations (see Supplementary Fig. S3).

**Figure 6:**
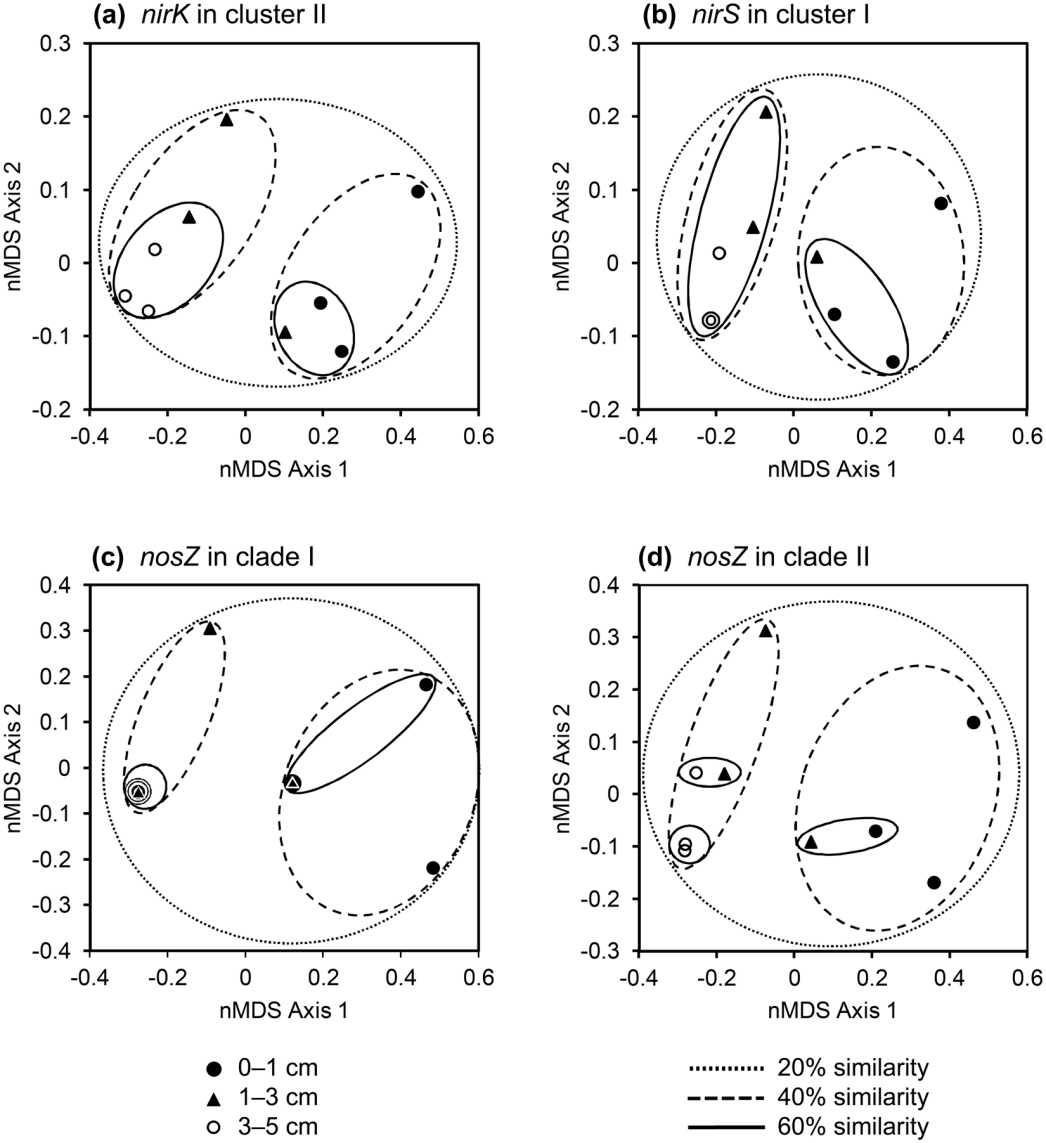
Nonmetric multidimensional scaling plot of Hellinger-transformed Bray-Curtis dissimilarity matrices describing microbial communities harboring *nirK* in cluster II (**a**), *nirS* in cluster I (**b**), *nosZ* in clade I (**c**), and *nosZ* in clade II (**d**) in −99-h soil cores. Overlapped symbols indicate equivalent values of data.

To determine the importance of soil depth directly, we presented the beta-diversities as nonmetric multidimensional scaling (nMDS) plots (Fig. 6). For each gene, all soil samples shared >20% similarity (Fig. 6). When grouping samples at 40% similarity (Fig. 6), soil samples could be grouped into two clusters: a cluster dominated by samples originating from surface soil (0–1 cm) and a cluster composed of samples from deeper soil (1–3 cm and 3–5 cm) (Fig. 6 and Supplementary Fig. S4), suggesting differences between surface and deeper soil in microbial communities. The microbial communities at soil depths of 3 to 5 cm showed high degrees of similarity and could be grouped together, even with similarity as high as 60% (Figs 6 and S4). Our results indicated that community composition of denitrifiers varied according to soil depth.

We then evaluated the identity of denitrifiers showing significant variations according to soil depth. For each gene, analysis using LEfSe algorithm^33^ identified OTUs enriched at each soil depth. Among these enriched OTUs, the dominant OTUs (OTU1–OTU20 for each gene) showing abundance >1% are summarized in Supplementary Table S11. Because these enriched OTUs were also dominant OTUs, the results presented in Supplementary Table S11 were almost in accord with data presented in Figs 3 and 4, except for OTUs affiliated with the genera *Bradyrhizobium* and *Melioribacter*, which covered multiple dominant OTUs.

### Effect of waterlogging on denitrifier communities

Because short-term waterlogging did not increase the copy numbers of denitrification genes at all examined soil depths (Fig. 2), despite the large episodic N_2_O peaks observed^17^, we determined whether the richness, biodiversity, and community composition of microbes harboring denitrification genes in soil changed in response to waterlogging. The richness and alpha-diversities of microbial communities between −99-h and 24-h soil cores did not exhibit significant differences (Supplementary Table S6). This result suggested that waterlogging had no effect on the biodiversity of the microbes harboring *nirK* in cluster II. To compare the microbial-community compositions between −99-h and 24-h soil cores, we performed multiple analyses, including analysis of molecular variance (AMOVA), homogeneity of molecular variance (HOMOVA), and weighted and unweighted UniFrac analyses as previously described^34^. Among these analyses, only weighted UniFrac showed significant differences in most comparisons (see Supplementary Table S12). According to a previous report^34^, these data indicated the presence of a pivot between the communities, i.e., the two communities shared a large portion of microbial species, although there were a portion of unique species in each community. To verify this hypothesis, we generated Venn diagrams (see Supplementary Fig. S5). Although some OTUs were not shared between −99-h and 24-h samples, for each gene, the shared OTUs exhibited relative abundance ranging from 94.3% to 99.7% at all soil depths, thus forming pivots between communities in −99-h and 24-h soil cores (see Supplementary Fig. S5). Either *nirS* in cluster I or *nosZ* in clade I exhibited higher relative abundance (≥99.0%) of shared OTUs in the −99-h and 24-h samples as compared with that observed for *nirK* in cluster II (94.3–98.3%) and *nosZ* in clade II (94.5–97.4%), suggesting that *nirS* in cluster I and *nosZ* in clade I exhibited weaker responses to waterlogging relative to those observed from *nirK* in cluster II and *nosZ* in clade II.

Despite differences in OTU identities, we then determined which OTU showed significant changes in abundance after waterlogging. Analysis using the LEfSe algorithm identified these OTUs at each soil depth. Among these OTUs, dominant OTUs (OTU1–OTU20 for each gene) showing significant changes in their abundance are summarized in Supplementary Table S13. Either *nirS* in cluster I or *nosZ* in clade I contained fewer significantly changed OTUs (five OTUs for *nirS* in cluster I and six OTUs for *nosZ* in clade I) as compared with *nirK* in cluster II (12 OTUs) and *nosZ* in clade II (10 OTUs), and the abundance of the most dominant OTUs (OTU1–OTU3) of *nirS* in cluster I and *nosZ* in clade I did not change significantly. These findings were consistent with those from the Venn diagrams, suggesting that the microbial communities harboring *nirS* in cluster I and *nosZ* in clade I showed weaker responses to waterlogging as compared with responses from communities harboring *nirK* in cluster II and *nosZ* in clade II, which are major players in the denitrification process in this soil.

## Discussion

Similar to other PCR-based detection methods, the reliability of pyrosequencing denitrification genes depends greatly upon the reliability of PCR primers. To collect intact and reliable information for denitrification genes from soil, the coverage for microbial-taxa sequences by PCR primers should be as high as possible. For this purpose, we used PCR primers targeting four clusters of *nirK*, three clusters of *nirS*, and two clades of *nosZ* to detect denitrifiers in soil. In our soil samples, both clades of *nosZ* were detected, whereas only one cluster of sequences was clearly detected for *nirK* and *nirS* (see Supplementary Fig. S1). Therefore, we used four sets of primers targeting these sequences to study the biodiversity of microbes harboring denitrification genes in soil.

Previous reports indicated positive correlations between *nirK* abundance and N_2_O emissions^20^ and negative correlations between *nirS* copy number and N_2_O/(N_2_O + N_2_) ratio^35^. Because the *nirK* copy number was higher than that of *nirS* in the soil used in this study (Fig. 2), we hypothesized that this soil exhibited high potential for releasing N_2_O during microbial denitrification. This hypothesis was supported by our previous observations in the field^15^ and soil-incubation experiments^17^, which showed sharp and explosive emissions of N_2_O from soil immediately after waterlogging.

According to a recent survey of microbial genomes harboring denitrification genes, the *nosZ* gene exhibits a higher frequency of co-occurrence with *nirS* as compared with *nirK* ^36^. Therefore, a higher ratio of *nirK*/*nirS* might suggest a lower abundance of the *nosZ* gene and a higher level of N_2_O emission. However, the *nosZ* gene in clade II does not co-occur with either *nirK* or *nirS* ^37^; therefore, the ratio of *nirK*/*nirS* may not always accurately predict the potential of soil to release N_2_O, particularly in clade II *nosZ*-dominated soils. Calculation of this ratio (*nosZ* in clades I and II)/(*nirK* + *nirS*) could account for *nosZ* in clade II and should constitute a better predictive approach. In this study, we observed no significant difference in copy numbers of denitrification genes after waterlogging. Therefore, the large N_2_O emissions observed in the upland converted paddy field after waterlogging^15^ may have been augmented by the low ratio of *nosZ* in clades I and II to *nirK* + *nirS* (~1:7 in the raw soil) in the soil rather than by the increased abundance of denitrifiers.

Using the newly developed PCR primers, we detected a wide range of microbial taxa in the soils. For the *nirK* gene, we detected sequences affiliated with 11 bacterial phyla/subphyla, as well as phyla of *Fungi* (*Ascomycetes*, grouped into the “Others” group) and *Protists* (*Amoebozoa*), with abundances of 0.1% and 4.4% in a total of 18 samples, respectively (Fig. 3a). This suggested that in this soil, aside from bacterial denitrifiers, *Fungi* and *Protists* may also contribute to denitrification, although this contribution might be minor. In contrast to the highly diverse sequences of the *nirK* gene, all of the dominant taxa associated with the denitrifiers harboring *nirS* in cluster I sequences belonged to the *Proteobacteria* phylum (Fig. 3b). Similar to the *nirS* gene, >99% of clade I sequences for the *nosZ* gene were also affiliated with the *Proteobacteria* phylum (Fig. 4a). The clade II sequences of the *nosZ* gene were affiliated with a wide range of bacterial and archaeal phyla (Fig. 4b), including 12 phyla/subphyla of bacteria and two phyla of archaea (*Crenarchaeota* and *Euryarchaeota*). These data were consistent with the primer design, because the primers targeting *nirS* in cluster I and *nosZ* in clade I mainly covered *alpha*-, *beta*-, and *gamma*-*proteobacteria* ^28, 29, 31^, whereas the primers targeting *nirK* in cluster II and *nosZ* in clade II covered a wide range of microbial phyla^28, 29^.

Distribution of microbial taxa according to soil depth was inconsistent. For microbes harboring *nirK* in cluster II, *Chthoniobacter* and *Ralstonia* were enriched in 0 to 1 cm of soil, whereas *Salinisphaera* and *Hyphomicrobium* were enriched in 3 to 5 cm of soil (Fig. 3a). Additionally, the copiotrophic taxon *Bacteroidetes* (*Flavihumibacter*, *Flavobacterium*, and *Niastella*) harboring *nosZ* in clade II was enriched in 0 to 1 cm of soil (Fig. 4b), which contained higher levels of nitrate-N as compared with deeper soil levels. This finding was consistent with that of a previous report indicating that soil with high levels of nitrogenous fertilizer favors a copiotrophic microbial community^38^.

The biogeographical distribution of soil microbial communities at the macroscale, including the continental scale^39–42^ and the field scale^43–50^, is well documented. Among the environmental factors investigated, soil pH is the most important factor in determining the biogeographical distribution of soil microbial communities^39–46, 51^. However, it is unclear whether soil pH is also the main factor at the microscale level (e.g., at the centimeter level). Here, we examined the effects of five environmental factors on the distribution of microbes harboring denitrification genes in soil at the microscale level, finding that soil depth, water-soluble carbon, and nitrate-N, but not soil pH, had significant effects on this distribution (Fig. 5 and Supplementary Table S10). Compared to the wide pH ranges spanning several pH units investigated at the continental and field scales, the pH range of our soil samples was narrow, spanning <0.5 pH units (see Supplementary Table S8). Therefore, it is possible that the effect of pH was too weak to be detected. Similar to our result, Yuan *et al*. recently reported that total nitrogen may be a primary factor that directly determines depth-related changes (0–20 cm of soil with pH ranges spanning 0.1–0.6 pH units) in soil bacterial-community composition^47^. Although further investigation using various types of soil is required, based on the available information, we believe that soil pH may not be the main factor at the microscale level for determining biogeographical distribution of soil microbial communities, given that pH ranges at this level may span <1 pH unit.

Microbial-community composition is often sensitive to the addition of nitrogen^52, 53^. Fierer *et al*. reported that at the field scale, long-term nitrogen fertilization had no significant effect on bacterial alpha-diversity, but a significant effect on bacterial-community composition^38^. However, the effect of soil nitrogen on microbes harboring denitrification genes at the microscale level was still unclear. In this study, we found that the alpha-diversity of microbes harboring the *nirK* gene in cluster II decreased along with the increases in soil depth to within 5 cm (see Supplementary Tables S6 and S7), and that this variation was positively correlated with water-soluble carbon, but was not correlated with nitrate-N (see Supplementary Table S9) levels, despite nitrate-N levels exhibiting a clear gradient based on soil depth in the analyzed samples. Additionally, by examining the effect of environmental factors on the beta-diversities of microbes harboring denitrification genes, we found that the beta-diversities of microbes harboring *nirK* in cluster II, *nirS* in cluster I, and *nosZ* in clades I and II were shaped by soil depth and water-soluble carbon, as well as nitrate-N (Fig. 5 and Supplementary Fig. S3). Moreover, we determined that the abundance of some OTUs, particularly dominant OTUs (see Supplementary Table S11), exhibited significant changes according to soil depth, suggesting variations in community composition based on soil depth. These findings indicated that soil nitrogen (nitrate-N) levels had no significant effect on the alpha-diversity of microbes harboring denitrification genes, but did exert a significant effect on beta-diversity and community composition, which were similar to findings associated with total bacterial composition in soil^38^.

According to a previous review discussing the creation and maintenance of microbial biogeographic patterns^54^, we hypothesized that selection and dispersal might be the two major processes shaping the beta-diversities of microbes harboring denitrification genes in soil. Because the water content of the field soil used in this study was low prior to waterlogging [water-filled pore space (WFPS) = 27 ± 7%], dispersal could be limited, which should have restricted microbial distribution. Selection eliminates microbes unable to adapt to local environments and should be reflected by differences in community compositions according to the gradients of nutrients, such as carbon and nitrogen. Therefore, a combination of nutrient selection and poor dispersal may have resulted in differences in microbial-community compositions. Because the level of both water-soluble carbon and nitrate-N showed clear gradients based on soil depth (see Supplementary Table S8), soil depth might be a proxy for various nutrients in soil as suggested in coastal ecosystems^55^. For each gene, our identification of different enriched OTUs at each soil depth (see Supplementary Table S11) might be explained as the result of selection and limited dispersal.

The field from which the soil samples used in this study originated is located in the East Asian monsoon region of Japan. In Japan, there are two rainy seasons from mid-May to late July and from mid-September to late October, with a dry period from late July to mid-September^56^. Even during the rainy season, rain and dry weather alternate frequently^15^. Therefore, from the beginning of the first rainy season to the end of the second rainy season, the field soil repeatedly experienced wet/dry cycles. Under such a climate, Akiyama *et al*. observed large N_2_O emissions following heavy rainfall from an upland converted paddy field^15^. Nitrous oxide emissions following heavy rainfall are common, and account for 55% to 80% of the annual N_2_O emission from this field^15^. Therefore, it was worth exploring the mechanisms underlying this phenomenon. As a result, we focused on the response of microbial communities, harboring denitrification genes, to short-term waterlogging.

The rewetting of dried soil upregulates expression of the *rpoB* gene, which encodes the β subunit of bacterial RNA polymerase, suggesting an active bacterial response to soil rewetting^57^. However, the active response of microbes in short-term waterlogged soil was unrelated to increased microbial populations as revealed by qPCR (Fig. 2), although the size of denitrifier community increased after a long-term soil wetting (one week or longer)^19^. Instead, we detected significant changes in microbial-community composition (see Supplementary Fig. S5 and Supplementary Tables S12 and S13). These data agreed with those from a previous report showing that simulated rainfall did not alter levels of microbial biomass carbon and total phospholipid fatty acid (PLFA), but did significantly alter microbial-community composition in two out of four soils according to PFLA profiles^58^.

In the soil samples examined in this study, water-soluble carbon was enriched at soil depths of 0 to 1 cm. According to a previous study^59^ and a review on irrigation^60^, this could be caused by repeated wet-dry cycles, which increase levels of dissolved organic carbon more quickly in surface soil than in deeper soil. The levels of nitrate-N were also higher at depths of 0 to 1 cm when compared with levels observed in deeper regions of these soil samples, which is a common phenomenon in soil subjected to a drying process^61, 62^. Because water-soluble carbon and nitrate-N were enriched at depths of 0 to 1 cm, N_2_O emissions from this surface soil should be greater than those from deeper soil as reported in previous studies^60, 63^. According to these results, we expected that the response to short-term waterlogging by rainfall would involve increased changes in the community composition of microbes harboring denitrification genes at soil depths of 0 to 1 cm relative to those observed in deeper soil. As shown in Supplementary Table S12, the weighted UniFrac distances for the microbial communities between the −99-h and 24-h samples were highest at soil depths of 0 to 1 cm, suggesting that the largest changes in microbial composition occurred in this region of the soil.

## Conclusion

Our study had some limitations. Although pyrosequencing of PCR amplicons is a powerful technique used to investigate microbial communities in soil, some pitfalls limiting its power could be introduced during the pre-requisite PCR process, which involve bias during amplification^64^. Furthermore, the primers previously used for PCR amplification of denitrification genes did not cover all possible sequences available in public databases^28, 29, 32, 37^. In order to detect as many of our target sequences as possible, we performed conventional PCR, qPCR, and pyrosequencing using recently developed PCR primers^28, 29, 31^. However, because the development of these new primers was based on collected sequence information from current databases, continuous updates to this sequence information will require frequent generation of new primers. From this viewpoint, we note that the detected sequence information in this study may not cover 100% of the sequences in nature. Additionally, we only examined one type of soil (Fluvisol) lacking aboveground plants. Because microbial-community responses to soil rewetting are modified in the presence of plants^56, 62^, it is necessary to examine other types of soil in the presence and absence of different aboveground plants to form a general consensus regarding their roles in this process. In this study, the denitrifier communities we detected represent the total community in the soil. To acquire the information of the active denitrifier communities responsive to waterlogging, sequencing of mRNA extracted from soil should be conducted.

In summary, our results indicated that the copy numbers of all denitrification genes examined did not change significantly according to soil depth and were unaffected by short-term waterlogging. We also found that the alpha-diversity of microbes harboring *nirK* in cluster II varied according to soil depth in soil cores prior to waterlogging (−99 h) and was positively correlated with levels of water-soluble carbon in the soil, whereas the beta-diversities of microbes harboring the *nirK*, *nirS*, and *nosZ* genes were affected by soil depth and water-soluble carbon, as well as nitrate-N levels. Furthermore, short-term waterlogging scarcely affected the abundance, richness, or alpha-diversities of microbes harboring denitrification genes, but had significant effects on their community compositions. Our results provided insight into the communities of microbes harboring *nirK*, *nirS*, and *nosZ* genes in soil ecosystems. Because short-term soil waterlogging causes large emissions of the greenhouse gas N_2_O and occurs frequently worldwide, these data could be helpful for investigating the mechanisms underlying N_2_O emissions caused by soil waterlogging and understanding the contribution of the denitrification process to the global nitrogen cycle.

## Methods

### Preparation of soil samples

Because our previous study showed that nitrate was dominantly distributed in the top soil and N_2_O was also mainly generated from the top soil^17^, we sampled soil cores to a depth of 5 cm beneath the ground’s surface in this study. Intact soil cores (100 cm^3^; 5-cm internal diameter, 5-cm length) were extracted from an experimental field at the Institute for Agro-Environmental Sciences, NARO, Japan (36.025° N, 140.107° E), on September 2012. The field was a former rice paddy that was tile-drained and had been used to grow soybeans for 6 years, followed by 1 year of being fallow. The soil in this field was a gray lowland soil (Fluvisol) and exhibited a water content of 27 ± 7% WFPS, a bulk density of 1.17 ± 0.10 Mg m^−3^, and was determined to be sandy clay loam soil according to the United States Department of Agriculture classification system^15^. During the experiments, all soil cores were incubated at 25 °C, at which the largest peak of N_2_O emission was observed in the field^15^, and short-term waterlogging was performed for 24 h, a period typical of a heavy rainfall event that occurs in the field. Addition of nitrate represented simulation of fertilizer management in the field and occurred at −3 h. One milliliter of KNO_3_ (9 g N L^−1^) was applied to the soil-core surface to achieve 9 mg N core^−1^, which corresponded to a rate of 46 kg N ha^−1^. During waterlogging, from 0 h to 24 h (Fig. 1a), the soil cores were water saturated by keeping the water table level no higher than the soil surface. Under this condition, an anaerobic condition was successfully established as reported previously^17^. Triplicate soil cores taken at −99 h (the raw soil), −3 h (prior to waterlogging), 3 h (shortly after commencement of waterlogging), 24 h (after completion of waterlogging), and 69 h (recovery from waterlogging) were used for DNA extraction (Fig. 1a). These soil cores were separated into three layers: 0 to 1 cm, 1 to 3 cm, and 3 to 5 cm (Fig. 1b). The cores were then snap-frozen in liquid nitrogen and stored at −80 °C until use.

### DNA extraction from soil

DNA was extracted from 0.4 g soil taken from each layer of the soil cores obtained at −99 h, −3 h, 3 h, 24 h, and 69 h of incubation (*n* = 3) using a FastDNA spin kit for soil (MP Biomedical, Santa Ana, CA, USA). To remove humic substances capable of potentially inhibiting enzymatic reactions in downstream analyses^65^, additional purification was performed using a MicroSpin S-400 HR column (GE Healthcare, Little Chalfont, UK) and a DNA clean & concentrator-5 kit (Zymo Research, Irvine, CA, USA). The concentrations of extracted DNA were measured using a NanoDrop ND-1000 UV-Vis spectrophotometer (Thermo Fisher Scientific, Waltham, MA, USA).

### Detection of denitrification genes using conventional and qPCR

Conventional PCR was performed to determine the existence of target sequences in DNA extracted from −99-h soil cores. A 25-μL reaction mixture contained 15 ng of soil DNA, primers (see Supplementary Table S1) at a final concentration of 0.2 μM (*nirS* and *nirK*) or 0.8 μM (*nosZ*), bovine serum albumin at a final concentrations of 0.48 mg mL^−1^ (*nirS* and *nirK*) or 1 mg mL^−1^ (*nosZ*), nuclease-free water, and the Premix Ex Taq polymerase (hot-start version; Takara Bio, Kusatsu, Shiga, Japan). Dimethyl sulfoxide was added to a final concentration of 5% to amplify cluster III sequences of *nirK*. A Veriti thermal cycler (Thermo Fisher Scientific) was used to perform conventional PCR. Cycling parameters for conventional PCR are listed in Supplementary Table S2.

The genes detected by conventional PCR in DNA extracted from −99-h soil cores were subjected to qPCR analysis using a QuantiTect SYBR Green PCR kit (Qiagen, Hilden, Germany) on a StepOnePlus Real-Time PCR system (Thermo Fisher Scientific). A 1-μL sample of 2-fold diluted soil DNA was used as a template in a 20-μL reaction mixture. The final concentration of each primer in the PCR mixture was 1 μM. Cycling parameters are listed in Supplementary Table S3.

### Pyrosequencing of PCR amplicons of *nirS* and *nosZ* genes

To investigate denitrifier communities in the soil before and after waterlogging, DNA extracted from the −99-h (raw soil, prior to waterlogging) and 24-h (immediately after waterlogging) soil cores was used to generate DNA fragments for pyrosequencing. Gene-specific primers are listed in Supplementary Table S4. A 26-nucleotide adapter, a 4-nucleotide key (TCAG), and a 10-nucleotide multiplex identifier (MID) tag (also known as a barcode, which is absent in the reverse primers) specific to each sample were successively added to the 5′-end of the pyrosequencing primer sequences. Pyrosequencing primers with different MID tags were screened *in silico* using OligoAnalyzer (http://sg.idtdna.com/calc/analyzer), followed by experimental verification. To reduce bias generated during PCR amplification, two-step PCR was performed according to a previous study^66^. One microliter of the first-step PCR-reaction result (20 cycles with the primers used in the conventional PCR) was used as the template for the second-step PCR (15 cycles with pyrosequencing primers). The PCR-cycling parameters were the same as those used for conventional PCR, except for the number of cycles. Each of the PCR products was purified using a PureLink PCR purification kit (Thermo Fisher Scientific), followed by further purification with agarose gels using a QIAquick gel-extraction kit (Qiagen), and verified as single sharp peaks according to high-sensitivity DNA assays using an Agilent 2100 Bioanalyzer system (Agilent Technologies, Santa Clara, CA, USA). After quantification using a TBS-380 mini-fluorimeter (Turner BioSystems, Sunnyvale, CA, USA) and a Quanti-iT PicoGreen dsDNA assay kit (Thermo Fisher Scientific), the purified PCR products for the same gene were diluted and mixed to equimolar ratios. Pyrosequencing of these purified PCR products on a GS Junior system (Roche, Basel, Switzerland) was conducted according to manufacturer protocol. A total of 72 DNA samples (three cores × three layers × two treatments × four genes) were sequenced using four Picotiter plates (one plate per run of the sequencer; Roche).

### Processing and analyzing pyrosequencing data

Pyrosequencing data were processed and analyzed using Mothur version 1.36.1^67^. The sequence reads were assigned to each sample according to sample-specific barcodes, with PCR primer and barcode sequences located at ends of the sequence reads subsequently trimmed. The criteria for retaining high-quality sequences were as follows: no base ambiguity, average quality score >25, homopolymer <8, one mismatch in the MID sequence, two mismatches in the primer sequence, minimum sequence lengths of 340 bp (*nirK* in cluster II), 350 bp (*nirS* in cluster I and *nosZ* in Clade I), and 420 bp (*nosZ* in clade II), and maximum sequence lengths of 460 bp (*nirK* in cluster II), 400 bp (*nirS* in cluster I), 450 bp (*nosZ* in clade I), and 540 bp (*nosZ* in clade II) (including the sequences of PCR primers and MIDs). The trimmed sequences were aligned against sequences downloaded from the FunGene database^68^. To reduce errors that may have occurred during pyrosequencing, 2% pre-clustering was performed as suggested^69^, and chimeric sequences identified by the UCHIME algorithm^70^ were removed. The remaining sequences were realigned using MAFFT^71^ and then clustered using the Furthest Neighbor algorithm, with cutoff values of 0.26 (*nirK* in cluster II), 0.17 (*nirS* in cluster I), and 0.18 (*nosZ* in clades I and II) corresponding to 74%, 83%, and 82% of the median similarity values of the denitrification gene sequences at the species level (see Supplementary Table S5), respectively, which were generated from ClustalW^72^ alignment of the sequences originating from the same genus. To acquire taxonomic identities of the OTUs, local BlastX^73^ searches against *nirK*, *nirS*, or *nosZ* sequences downloaded from the FunGene database were performed using the representative sequences of each OTU as queries.

To correct differences in sequencing depth among soil samples^74^, alpha- and beta-diversity analyses of each gene were performed using a randomly selected subset of 1841 (*nirK* cluster II), 5855 (*nirS* cluster I), 6281 (*nosZ* clade I), and 1826 (*nosZ* clade II) sequences per soil sample (the minimum number of sequences recovered among 18 samples per gene). Beta-diversities were presented as nMDS plots, which were generated from Hellinger-transformed Bray-Curtis matrices using the Vegan package in R (v3.2.5; http://www.R-project.org/), and Venn diagrams, which were generated from the VennDiagram package (v1.6.9)^75^ in R. CCA and variation-partition analyses were performed using the Vegan package in R to identify important environmental factors and their contributions to variations in microbial communities. LEfSe^33^, which was implemented in Mothur, was used to examine which OTU showed significantly varied abundance among samples.

### Statistical analyses

One-way ANOVA and Tukey’s post-test were performed using R to examine variations in microbial richness and alpha-diversity indices. Spearman’s rank correlation test was performed using R to explore correlations between the alpha-diversity indices of the microbes harboring *nirK* in cluster II and environmental factors. An ANOVA-like permutation test and PERMANOVA were performed using the Vegan package in R to examine the significance of CCA results. Mothur was also used to test the global and pairwise differences among sample groups according to AMOVA^76^ and HOMOVA^77^, as well as weighted- and unweighted UniFrac analyses^78^.

### Deposition of DNA sequences

All sequences acquired from pyrosequencing in this study were deposited to the DDBJ Sequence Read Archive under accession number DRA005237.

## Electronic supplementary material

Supplementary information
