# Supplementary material for: Responses of denitrifying bacterial communities to short-term waterlogging of soils
Source: Sci Rep. 2017 Apr 11;7:803. doi: 10.1038/s41598-017-00953-8 (PMC5429771; doi:10.1038/s41598-017-00953-8)
Supplement: Supplementary file 1 — Supplementary information [file 41598_2017_953_MOESM1_ESM.pdf]

Supplementary information

Responses of denitrifying bacterial communities to short-term waterlogging of soils

Yong Wang[1,†,*], Yoshitaka Uchida[2,†], Yumi Shimomura[1,#], Hiroko Akiyama[1] & Masahito Hayatsu[1,*]

[1]Institute for Agro-Environmental Sciences, National Agriculture and Food Research Organization (NARO), 3-1-3, Kannondai, Tsukuba, Ibaraki 305-8604, Japan. [2]Research Faculty of Agriculture, Hokkaido University, Kita 9, Nishi 9, Kita-ku, Sapporo, Hokkaido, 060-8589, Japan. [†]These authors contributed equally to this work. [#]Present address: Kyodo Milk Industry Co., Ltd, 20-1, Hirai, Hinode, Nishitama, Tokyo 190-0182, Japan. [*]Correspondence and requests for materials should be addressed to Y.W. (email: ywangbioinfo@gmail.com) or M.H. (email: hayatsu@affrc.go.jp)

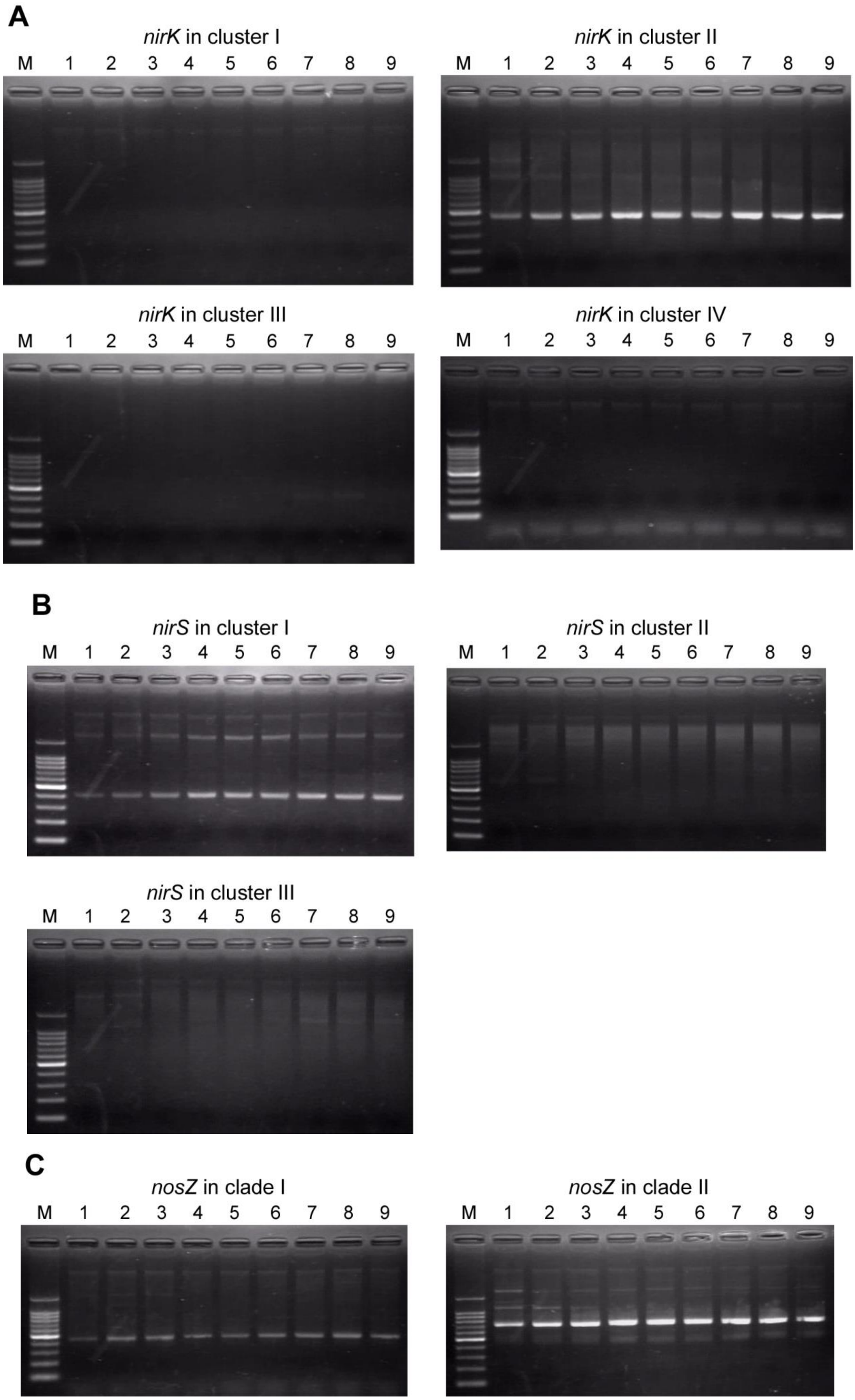
A
nirK in cluster I
M 1 2 3 4 5 6 7 8 9
nirK in cluster II
M 1 2 3 4 5 6 7 8 9
nirK in cluster III
M 1 2 3 4 5 6 7 8 9
nirK in cluster IV
M 1 2 3 4 5 6 7 8 9
B
nirS in cluster I
M 1 2 3 4 5 6 7 8 9
nirS in cluster II
M 1 2 3 4 5 6 7 8 9
nirS in cluster III
M 1 2 3 4 5 6 7 8 9
C
nosZ in clade I
M 1 2 3 4 5 6 7 8 9
nosZ in clade II
M 1 2 3 4 5 6 7 8 9

Supplementary Figure S1. Agarose gel electrophoretic analyses of the conventional PCR products amplified using the primers for all types of *nirK* (A), *nirS* (B), and *nosZ* (C) genes. M, molecular-size marker (100-bp DNA ladder; Toyobo, Osaka, Japan); lanes 1–3, results from DNA extracted at depths of 0 to 1 cm from soil-core samples; lanes 4–6, results from DNA extracted at depths of 1 to 3 cm from soil-core samples; lanes 7–9, results from DNA extracted at depths of 3 to 5 cm from soil-core samples. All samples were analyzed in triplicate.

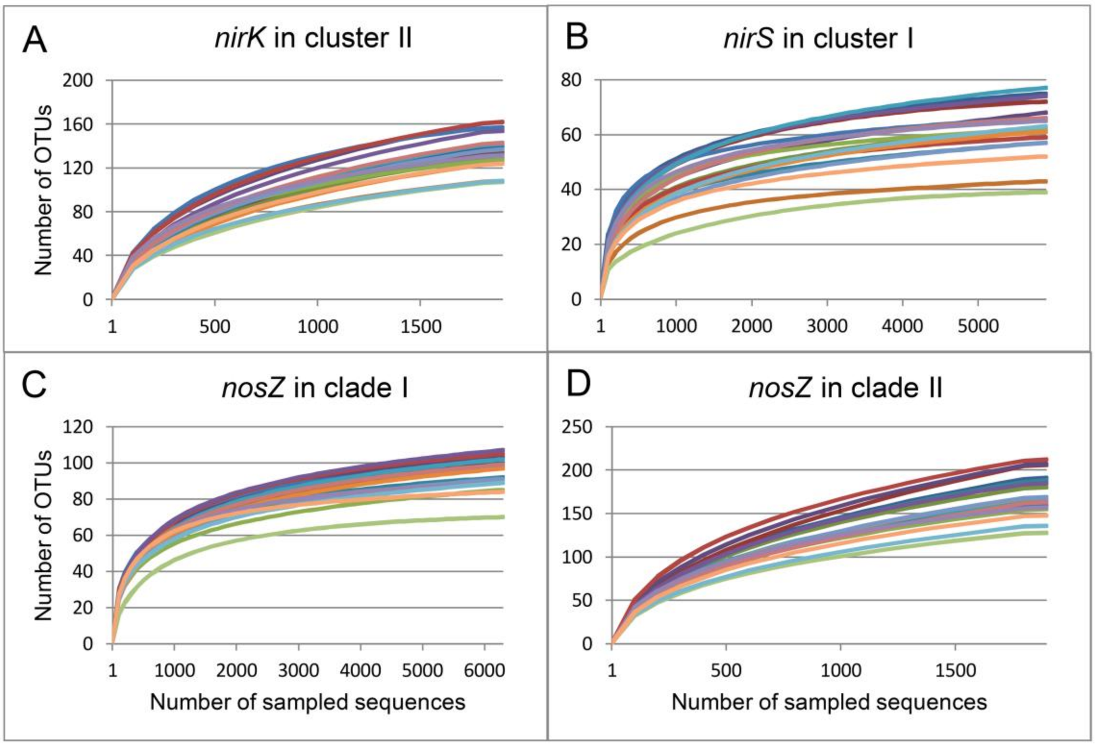

Supplementary Figure S2. The rarefaction curves for *nirK* in cluster II (A), *nirS* in cluster I (B), *nosZ* in clade I (C), and *nosZ* in clade II (D) sequences.

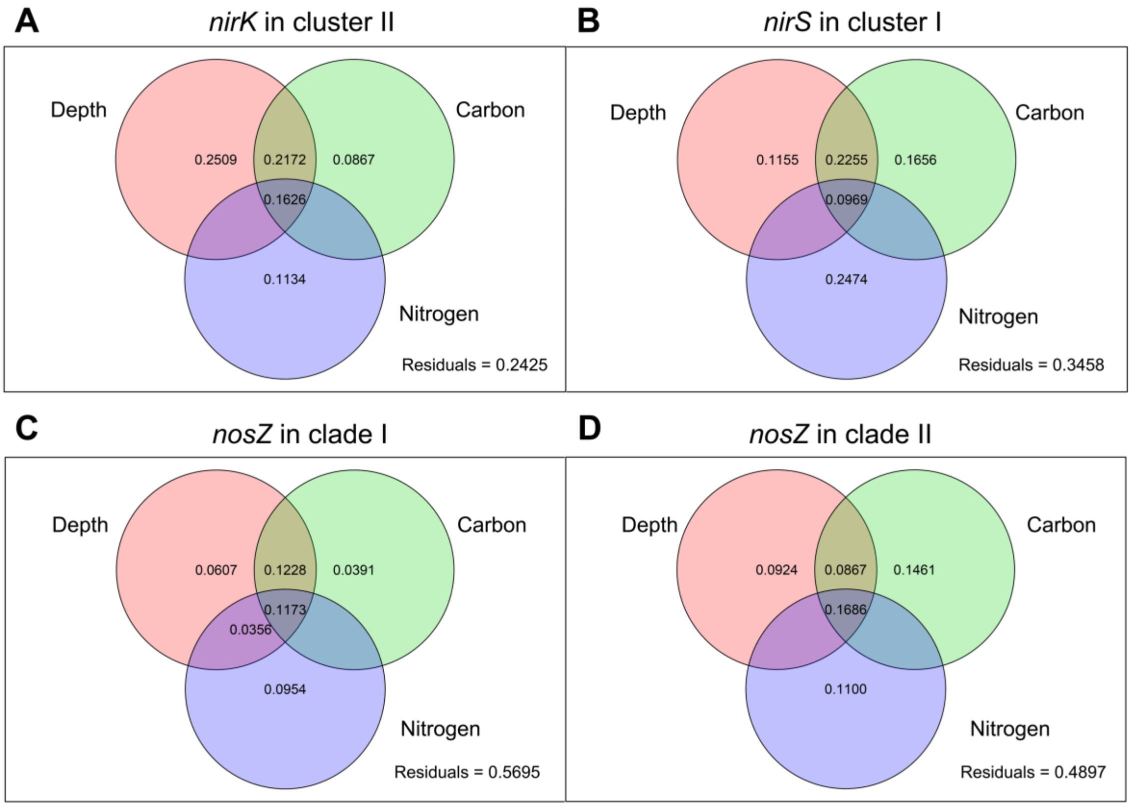

Supplementary Figure S3. Venn diagrams showing the contributions of soil depth, water-soluble carbon, and nitrate-N on the observed beta-diversity of bacteria via the calculation of variation partitions for *nirK* in cluster II (A), *nirS* in cluster I (B), *nosZ* in clade I (C), and *nosZ* in clade II (D). Negative values are not shown.

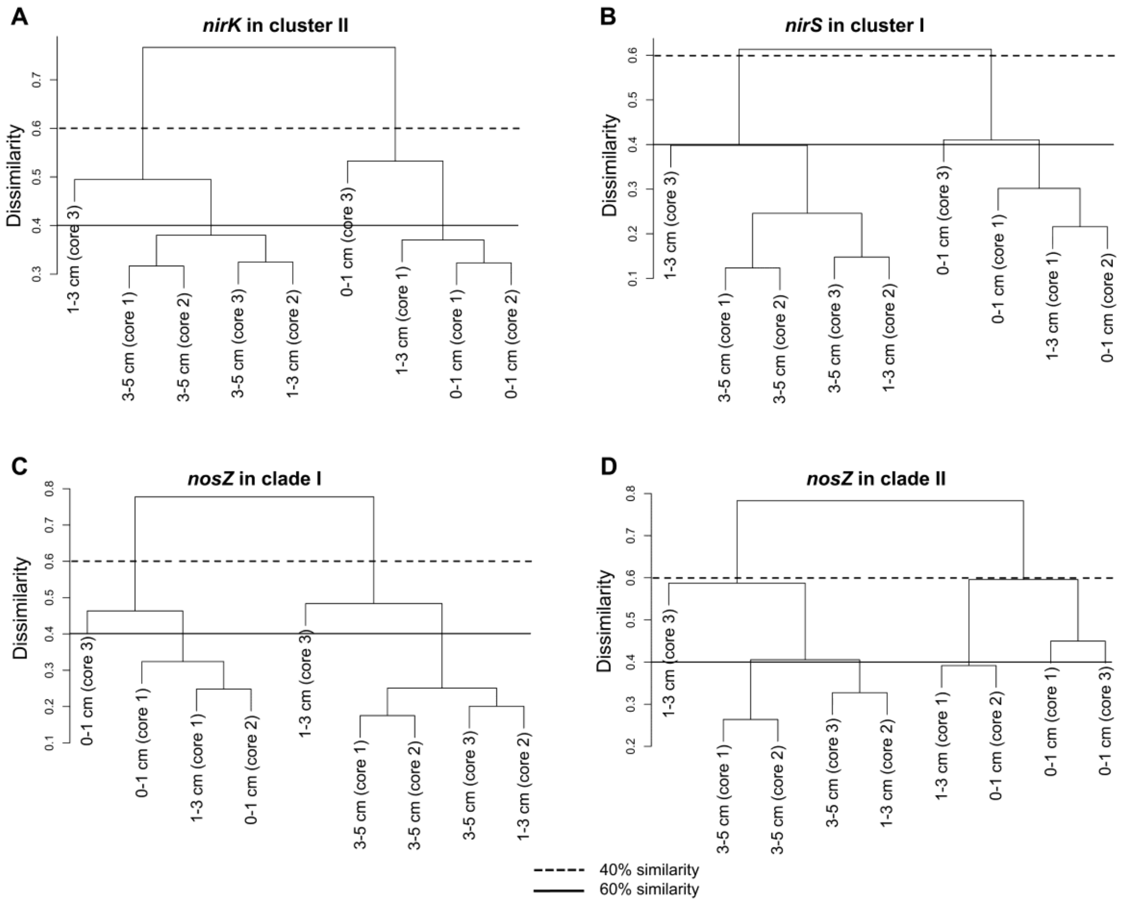

Supplementary Figure S4. Dendrograms based on hierarchical-clustering analysis of the denitrifier communities using Hellinger-transformed Bray-Curtis dissimilarity matrices for *nirK* in cluster II (A), *nirS* in cluster I (B), *nosZ* in clade I (C), and *nosZ* in clade II (D).

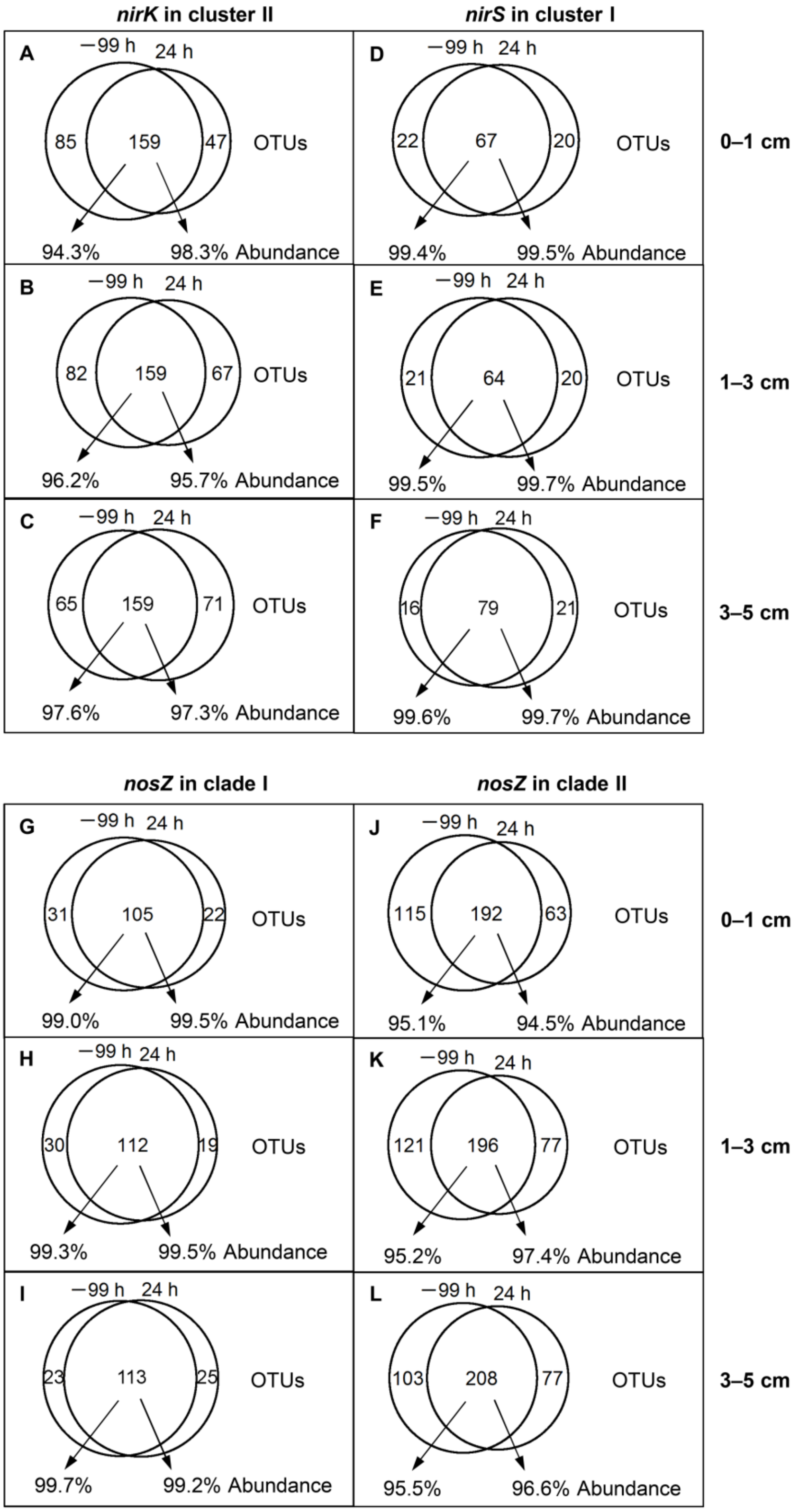

Supplementary Figure S5. Venn diagrams showing the existence of pivots in denitrifier communities harboring *nirK* in cluster II (A, B, and C), *nirS* in cluster I (D, E, and F), *nosZ* in clade I (G, H, and I), and *nosZ* in clade II (J, K, and L) before (−99 h) and after (24 h) waterlogging. The digits inside the circles indicate the number of OTUs, and the percentages indicate the relative abundance of OTUs shared by the communities before and after waterlogging.

Supplementary Table S1. Primers used for conventional and quantitative PCR

Genes	*Cluster/ Clade*	*Primer orientation*	*Primer IDs*	*Nucleotide sequences (5′→3′)*	*Annealing temperature (°C)*	*References*
nirK	I	Forward	nirKC1F	ATGGCGCCATCATGGTNYTNCC	54	Wei et al., 2015
nirK	I	Reverse	nirKC1R	TCGAAGGCCTCGATNARRTTRTG		
nirK	II	Forward	nirKC2F	TGCACATCGCCAACGGNATGTWYGG	56	Wei et al., 2015
nirK	II	Reverse	nirKC2R	GGCGCGGAAGATGSHRTGRTCNAC		
nirK	III	Forward	nirKC3F	CATCGGCAACGGCATGYAYGGNGC	58	Wei et al., 2015
nirK	III	Reverse	nirKC3R	CGACCATGGCCGTGGSWNACRAANGG		
nirK	IV	Forward	nirKC4F	TACGGTGTGATCATCRTSGATCC	60	Wei et al., 2015
nirK	IV	Reverse	nirKC4R	GCATCACGCATGGAATGATYSAC		
nirS	I	Forward	nirSC1F	ATCGTCAACGTCAARGARACVGG	56	Wei et al., 2015
nirS	I	Reverse	nirSC1R	TTCGGGTGCGTCTTSABGAASAG		
nirS	II	Forward	nirSC2F	TGGAGAACGCCGGNCARGTNTGG	56	Wei et al., 2015
nirS	II	Reverse	nirSC2R	GATGATGTCCACGGCNACRTANGG		
nirS	III	Forward	nirSC3F	TTCGCCCTGAARGAYGGNGG	56	Wei et al., 2015
nirS	III	Reverse	nirSC3R	AGGTGCCCACGAANARNCCNCC		
nosZ	I	Forward	nosZC1F	CGCTSTTYMTIGAYAGYCAG	54	Jones et al. 2014
nosZ	I	Reverse	nosZC1R	SKSACCTTITTRCCITYICG		
nosZ	II	Forward	nosZC2F	CTIGGICCIYTKCAYAC	54	Jones et al. 2014
nosZ	II	Reverse	nosZC2R	GCIGARCARAAITCBGTRC		

Supplementary Table S2. Cycling parameters used for conventional PCR

Cycling parameters	*nirK in cluster I*	*nirK in cluster II*	*nirK in cluster III*	*nirK in cluster IV*	*nirS*	*nosZ*
Activation of enzyme	95°C, 15 s	95°C, 15 s	98°C, 15 s	95°C, 15 s	95°C, 15 s	95°C, 15 s
Cycling (30 cycles)						
Denature	95°C, 30 s	95°C, 30 s	98°C, 30 s	95°C, 30 s	95°C, 30 s	95°C, 30 s
Annealing	54°C, 30 s	56°C, 30 s	58°C, 30 s	60°C, 30 s	56°C, 30 s	54°C, 1 min
Extension	72°C, 30 s	72°C, 30 s	72°C, 30 s	72°C, 30 s	72°C, 30 s	72°C, 1 min
Additional extension	72°C, 10 min	72°C, 10 min	72°C, 10 min	72°C, 10 min	72°C, 10 min	72°C, 10 min

Supplementary Table S3. Cycling parameters used for quantitative PCR

Cycling parameters	*nirK* in cluster II and *nirS* in cluster I	*nosZ* in clade I and II
Activation of enzyme	95°C, 15 min	95°C, 15 min
Cycling (40 cycles)		
Denature	95°C, 15 s	95°C, 15 s
Annealing	56°C, 30 s	54°C, 1 min
Extension	72°C, 30 s	72°C, 1 min
Detection of signal	82°C, 10 s	82°C, 10 s
	95°C, 15 s	95°C, 15 s
Melting curve	60°C, 1 min	60°C, 1 min
	95°C, 15 s	95°C, 15 s

Supplementary Table S4. Barcoded PCR primers used for pyrosequencing

Genes	*MIDs*	*Primer orientation*	*Soil samples*		*Primer IDs*	*Nucleotide sequences (5'→3')*
			Soil depths	*Soil core*		
nirK in cluster II		Reverse			nirKCII-R	CCTATCCCCTGTGTGCCTTGGCAGTCTCAGGGCGCGGAAGATGSHRTGRTCNAC
	MID11	Forward	0-1 cm	1	nirKCII-F-MID11	CCATCTCATCCCTGCGTGTCTCCGACTCAGTGATACGTCTTGCACATCGCCAACGGNATGTWYGG
	MID15	Forward	0-1 cm	2	nirKCII-F-MID15	CCATCTCATCCCTGCGTGTCTCCGACTCAGATACGACGTATGCACATCGCCAACGGNATGTWYGG
	MID16	Forward	0-1 cm	3	nirKCII-F-MID16	CCATCTCATCCCTGCGTGTCTCCGACTCAGTCACGTACTATGCACATCGCCAACGGNATGTWYGG
	MID17	Forward	1-3 cm	1	nirKCII-F-MID17	CCATCTCATCCCTGCGTGTCTCCGACTCAGCGTCTAGTACTGCACATCGCCAACGGNATGTWYGG
	MID19	Forward	1-3 cm	2	nirKCII-F-MID19	CCATCTCATCCCTGCGTGTCTCCGACTCAGTGTACTACTCTGCACATCGCCAACGGNATGTWYGG
	MID28	Forward	1-3 cm	3	nirKCII-F-MID28	CCATCTCATCCCTGCGTGTCTCCGACTCAGACTACTATGTTGCACATCGCCAACGGNATGTWYGG
	MID30	Forward	3-5 cm	1	nirKCII-F-MID30	CCATCTCATCCCTGCGTGTCTCCGACTCAGAGACTATACTTGCACATCGCCAACGGNATGTWYGG
	MID31	Forward	3-5 cm	2	nirKCII-F-MID31	CCATCTCATCCCTGCGTGTCTCCGACTCAGAGCGTCGTCTTGCACATCGCCAACGGNATGTWYGG
	MID32	Forward	3-5 cm	3	nirKCII-F-MID32	CCATCTCATCCCTGCGTGTCTCCGACTCAGAGTACGCTATTGCACATCGCCAACGGNATGTWYGG
	MID33	Forward	0-1 cm	1	nirKCII-F-MID33	CCATCTCATCCCTGCGTGTCTCCGACTCAGATAGAGTACTTGCACATCGCCAACGGNATGTWYGG
	MID37	Forward	0-1 cm	2	nirKCII-F-MID37	CCATCTCATCCCTGCGTGTCTCCGACTCAGTACACACACTTGCACATCGCCAACGGNATGTWYGG
	MID40	Forward	0-1 cm	3	nirKCII-F-MID40	CCATCTCATCCCTGCGTGTCTCCGACTCAGTACGCTGTCTTGCACATCGCCAACGGNATGTWYGG
	MID41	Forward	1-3 cm	1	nirKCII-F-MID41	CCATCTCATCCCTGCGTGTCTCCGACTCAGTAGTGTAGATTGCACATCGCCAACGGNATGTWYGG
	MID45	Forward	1-3 cm	2	nirKCII-F-MID45	CCATCTCATCCCTGCGTGTCTCCGACTCAGTCTATACTATTGCACATCGCCAACGGNATGTWYGG
	MID46	Forward	1-3 cm	3	nirKCII-F-MID46	CCATCTCATCCCTGCGTGTCTCCGACTCAGTGACGTATGTTGCACATCGCCAACGGNATGTWYGG
	MID47	Forward	3-5 cm	1	nirKCII-F-MID47	CCATCTCATCCCTGCGTGTCTCCGACTCAGTGTGAGTAGTTGCACATCGCCAACGGNATGTWYGG
	MID48	Forward	3-5 cm	2	nirKCII-F-MID48	CCATCTCATCCCTGCGTGTCTCCGACTCAGACAGTATATATGCACATCGCCAACGGNATGTWYGG
	MID50	Forward	3-5 cm	3	nirKCII-F-MID50	CCATCTCATCCCTGCGTGTCTCCGACTCAGACTAGCAGTATGCACATCGCCAACGGNATGTWYGG

		Reverse			nirSCI-R	CCTATCCCCTGTGTGCCTTGGCAGTCTCAGTTCGGGTGCGTCTTSABGAASAG
	MID4	Forward	0-1 cm	1	nirSCI-F-MID4	CCATCTCATCCCTGCGTGTCTCCGACTCAGAGCACTGTAGATCGTCAACGTCAARGARACVGG
	MID11	Forward	0-1 cm	2	nirSCI-F-MID11	CCATCTCATCCCTGCGTGTCTCCGACTCAGTGATACGTCTATCGTCAACGTCAARGARACVGG
	MID13	Forward	0-1 cm	3	nirSCI-F-MID13	CCATCTCATCCCTGCGTGTCTCCGACTCAGCATAGTAGTGATCGTCAACGTCAARGARACVGG
	MID14	Forward	1-3 cm	1	nirSCI-F-MID14	CCATCTCATCCCTGCGTGTCTCCGACTCAGCGAGAGATACATCGTCAACGTCAARGARACVGG
	MID17	Forward	1-3 cm	2	nirSCI-F-MID17	CCATCTCATCCCTGCGTGTCTCCGACTCAGCGTCTAGTACATCGTCAACGTCAARGARACVGG
	MID19	Forward	1-3 cm	3	nirSCI-F-MID19	CCATCTCATCCCTGCGTGTCTCCGACTCAGTGTACTACTCATCGTCAACGTCAARGARACVGG
	MID21	Forward	3-5 cm	1	nirSCI-F-MID21	CCATCTCATCCCTGCGTGTCTCCGACTCAGCGTAGACTAGATCGTCAACGTCAARGARACVGG
	MID22	Forward	3-5 cm	2	nirSCI-F-MID22	CCATCTCATCCCTGCGTGTCTCCGACTCAGTACGAGTATGATCGTCAACGTCAARGARACVGG
nirS in cluster I	MID28	Forward	3-5 cm	3	nirSCI-F-MID28	CCATCTCATCCCTGCGTGTCTCCGACTCAGACTACTATGTATCGTCAACGTCAARGARACVGG
	MID30	Forward	0-1 cm	1	nirSCI-F-MID30	CCATCTCATCCCTGCGTGTCTCCGACTCAGAGACTATACTATCGTCAACGTCAARGARACVGG
	MID31	Forward	0-1 cm	2	nirSCI-F-MID31	CCATCTCATCCCTGCGTGTCTCCGACTCAGAGCGTCGTCTATCGTCAACGTCAARGARACVGG
	MID37	Forward	0-1 cm	3	nirSCI-F-MID37	CCATCTCATCCCTGCGTGTCTCCGACTCAGTACACACACTATCGTCAACGTCAARGARACVGG
	MID39	Forward	1-3 cm	1	nirSCI-F-MID39	CCATCTCATCCCTGCGTGTCTCCGACTCAGTACAGATCGTATCGTCAACGTCAARGARACVGG
	MID44	Forward	1-3 cm	2	nirSCI-F-MID44	CCATCTCATCCCTGCGTGTCTCCGACTCAGTCTAGCGACTATCGTCAACGTCAARGARACVGG
	MID45	Forward	1-3 cm	3	nirSCI-F-MID45	CCATCTCATCCCTGCGTGTCTCCGACTCAGTCTATACTATATCGTCAACGTCAARGARACVGG
	MID47	Forward	3-5 cm	1	nirSCI-F-MID47	CCATCTCATCCCTGCGTGTCTCCGACTCAGTGTGAGTAGTATCGTCAACGTCAARGARACVGG
	MID48	Forward	3-5 cm	2	nirSCI-F-MID48	CCATCTCATCCCTGCGTGTCTCCGACTCAGACAGTATATAATCGTCAACGTCAARGARACVGG
	MID50	Forward	3-5 cm	3	nirSCI-F-MID50	CCATCTCATCCCTGCGTGTCTCCGACTCAGACTAGCAGTAATCGTCAACGTCAARGARACVGG

		Reverse			nosZCI-R	CCTATCCCCTGTGTGCCTTGGCAGTCTCAGSKSACCTTITTRCCITYICG
nosZ in clade I	MID1	Forward	0-1 cm	1	nosZCI-F-MID1	CCATCTCATCCCTGCGTGTCTCCGACTCAGACGAGTGCGTCGCTSTTYMTIGAYAGYCAG
	MID11	Forward	0-1 cm	2	nosZCI-F-MID11	CCATCTCATCCCTGCGTGTCTCCGACTCAGTGATACGTCTCGCTSTTYMTIGAYAGYCAG
	MID16	Forward	0-1 cm	3	nosZCI-F-MID16	CCATCTCATCCCTGCGTGTCTCCGACTCAGTCACGTACTACGCTSTTYMTIGAYAGYCAG
	MID19	Forward	1-3 cm	1	nosZCI-F-MID19	CCATCTCATCCCTGCGTGTCTCCGACTCAGTGTACTACTCCGCTSTTYMTIGAYAGYCAG
	MID28	Forward	1-3 cm	2	nosZCI-F-MID28	CCATCTCATCCCTGCGTGTCTCCGACTCAGACTACTATGTCGCTSTTYMTIGAYAGYCAG
	MID30	Forward	1-3 cm	3	nosZCI-F-MID30	CCATCTCATCCCTGCGTGTCTCCGACTCAGAGACTATACTCGCTSTTYMTIGAYAGYCAG
	MID32	Forward	3-5 cm	1	nosZCI-F-MID32	CCATCTCATCCCTGCGTGTCTCCGACTCAGAGTACGCTATCGCTSTTYMTIGAYAGYCAG
	MID37	Forward	3-5 cm	2	nosZCI-F-MID37	CCATCTCATCCCTGCGTGTCTCCGACTCAGTACACACACTCGCTSTTYMTIGAYAGYCAG
	MID39	Forward	3-5 cm	3	nosZCI-F-MID39	CCATCTCATCCCTGCGTGTCTCCGACTCAGTACAGATCGTCGCTSTTYMTIGAYAGYCAG
	MID40	Forward	0-1 cm	1	nosZCI-F-MID40	CCATCTCATCCCTGCGTGTCTCCGACTCAGTACGCTGTCTCGCTSTTYMTIGAYAGYCAG
	MID41	Forward	0-1 cm	2	nosZCI-F-MID41	CCATCTCATCCCTGCGTGTCTCCGACTCAGTAGTGTAGATCGCTSTTYMTIGAYAGYCAG
	MID42	Forward	0-1 cm	3	nosZCI-F-MID42	CCATCTCATCCCTGCGTGTCTCCGACTCAGTCGATCACGTCGCTSTTYMTIGAYAGYCAG
	MID45	Forward	1-3 cm	1	nosZCI-F-MID45	CCATCTCATCCCTGCGTGTCTCCGACTCAGTCTATACTATCGCTSTTYMTIGAYAGYCAG
	MID46	Forward	1-3 cm	2	nosZCI-F-MID46	CCATCTCATCCCTGCGTGTCTCCGACTCAGTGACGTATGTCGCTSTTYMTIGAYAGYCAG
	MID50	Forward	1-3 cm	3	nosZCI-F-MID50	CCATCTCATCCCTGCGTGTCTCCGACTCAGACTAGCAGTACGCTSTTYMTIGAYAGYCAG
	MID52	Forward	3-5 cm	1	nosZCI-F-MID52	CCATCTCATCCCTGCGTGTCTCCGACTCAGAGTATACATACGCTSTTYMTIGAYAGYCAG
	MID54	Forward	3-5 cm	2	nosZCI-F-MID54	CCATCTCATCCCTGCGTGTCTCCGACTCAGAGTGCTACGACGCTSTTYMTIGAYAGYCAG
	MID60	Forward	3-5 cm	3	nosZCI-F-MID60	CCATCTCATCCCTGCGTGTCTCCGACTCAGCTACGCTCTACGCTSTTYMTIGAYAGYCAG

		Reverse			nosZCII-R	CCTATCCCCTGTGTGCCTTGGCAGTCTCAGGCIGARCARAAITCBGTRC
	MID4	Forward	0-1 cm	1	nosZCII-F-MID4	CCATCTCATCCCTGCGTGTCTCCGACTCAGAGCACTGTAGCTIGGICCIYTKCAYAC
	MID11	Forward	0-1 cm	2	nosZCII-F-MID11	CCATCTCATCCCTGCGTGTCTCCGACTCAGTGATACGTCTCTIGGICCIYTKCAYAC
	MID13	Forward	0-1 cm	3	nosZCII-F-MID13	CCATCTCATCCCTGCGTGTCTCCGACTCAGCATAGTAGTGCTIGGICCIYTKCAYAC
	MID14	Forward	1-3 cm	1	nosZCII-F-MID14	CCATCTCATCCCTGCGTGTCTCCGACTCAGCGAGAGATACCTIGGICCIYTKCAYAC
	MID15	Forward	1-3 cm	2	nosZCII-F-MID15	CCATCTCATCCCTGCGTGTCTCCGACTCAGATACGACGTACTIGGICCIYTKCAYAC
	MID16	Forward	1-3 cm	3	nosZCII-F-MID16	CCATCTCATCCCTGCGTGTCTCCGACTCAGTCACGTACTACTIGGICCIYTKCAYAC
	MID17	Forward	3-5 cm	1	nosZCII-F-MID17	CCATCTCATCCCTGCGTGTCTCCGACTCAGCGTCTAGTACCTIGGICCIYTKCAYAC
	MID19	Forward	3-5 cm	2	nosZCII-F-MID19	CCATCTCATCCCTGCGTGTCTCCGACTCAGTGTACTACTCCTIGGICCIYTKCAYAC
nosZ in clade II	MID20	Forward	3-5 cm	3	nosZCII-F-MID20	CCATCTCATCCCTGCGTGTCTCCGACTCAGACGACTACAGCTIGGICCIYTKCAYAC
	MID21	Forward	0-1 cm	1	nosZCII-F-MID21	CCATCTCATCCCTGCGTGTCTCCGACTCAGCGTAGACTAGCTIGGICCIYTKCAYAC
	MID22	Forward	0-1 cm	2	nosZCII-F-MID22	CCATCTCATCCCTGCGTGTCTCCGACTCAGTACGAGTATGCTIGGICCIYTKCAYAC
	MID23	Forward	0-1 cm	3	nosZCII-F-MID23	CCATCTCATCCCTGCGTGTCTCCGACTCAGTACTCTCGTGCTIGGICCIYTKCAYAC
	MID24	Forward	1-3 cm	1	nosZCII-F-MID24	CCATCTCATCCCTGCGTGTCTCCGACTCAGTAGAGACGAGCTIGGICCIYTKCAYAC
	MID28	Forward	1-3 cm	2	nosZCII-F-MID28	CCATCTCATCCCTGCGTGTCTCCGACTCAGACTACTATGTCTIGGICCIYTKCAYAC
	MID30	Forward	1-3 cm	3	nosZCII-F-MID30	CCATCTCATCCCTGCGTGTCTCCGACTCAGAGACTATACTCTIGGICCIYTKCAYAC
	MID31	Forward	3-5 cm	1	nosZCII-F-MID31	CCATCTCATCCCTGCGTGTCTCCGACTCAGAGCGTCGTCTCTIGGICCIYTKCAYAC
	MID32	Forward	3-5 cm	2	nosZCII-F-MID32	CCATCTCATCCCTGCGTGTCTCCGACTCAGAGTACGCTATCTIGGICCIYTKCAYAC
	MID36	Forward	3-5 cm	3	nosZCII-F-MID36	CCATCTCATCCCTGCGTGTCTCCGACTCAGCGACGTGACTCTIGGICCIYTKCAYAC

Supplementary Table S5. Median similarity values associated with denitrification-gene nucleotide sequences at the species level

nirK in cluster II		*nirS in cluster I*		*nosZ in clade I*		*nosZ in clade II*	
Genera	*Median similarity*	*Genera*	*Median similarity*	*Genera*	*Median similarity*	*Genera*	*Median similarity*
Brucella	99%	*Comamonas*	97%	*Brucella*	100%	*Brucella*	100%
Ensifer	99%	*Ralstonia*	90%	*Rhodanobacter*	96%	*Burkholderia*	96%
Ochrobactrum	98%	*Halomonas*	89%	*Burkholderia*	95%	*Rhodanobacter*	96%
Achromobacter	89%	*Thermus*	88%	*Anaeromyxobacter*	93%	*Haloarcula*	94%
Rhodanobacter	89%	*Cupriavidus*	85%	*Haloarcula*	93%	*Halomonas*	94%
Crenarchaeote	88%	*Paracoccus*	85%	*Halomonas*	92%	*Geobacillus*	91%
Neisseria	88%	*Acidovorax*	**83%**	*Geobacillus*	91%	*Leisingera*	90%
Rhodopseudomonas	86%	*Marinobacter*	82%	*Neisseria*	89%	*Neisseria*	90%
Haloferax	83%	*Azoarcus*	74%	*Ralstonia*	89%	*Ralstonia*	89%
Burkholderia	82%	*Magnetospirillum*	74%	*Capnocytophaga*	88%	*Desulfitobacterium*	87%
Mesorhizobium	81%	*Accumulibacter*	73%	*Halorubrum*	88%	*Magnetospirillum*	87%
Sinorhizobium	81%	*Labrenzia*	60%	*Desulfitobacterium*	86%	*Acidovorax*	84%
Alcaligenes	80%	*Ruegeria*	60%	*Haloferax*	86%	*Labrenzia*	84%
Rhizobium	80%			*Acidovorax*	84%	*Marinobacter*	84%
Kingella	77%			*Labrenzia*	84%	*Pseudomonas*	84%
Capnocytophaga	75%			*Marinobacter*	84%	*Thioalkalivibrio*	84%
Paracoccus	75%			*Pseudomonas*	84%	*Azospirillum*	83%
Pusillimonas	75%			*Thioalkalivibrio*	83%	*Natronococcus*	83%
Actinobacillus	**74%**			*Bradyrhizobium*	82%	*Rhodobacter*	82%
Haloarcula	72%			*Natronococcus*	**82%**	*Vibrio*	**82%**

Bosea	71%	*Rhodobacter*	82%	*Bradyrhizobium*	81%
Corynebacterium	69%	*Ruegeria*	82%	*Capnocytophaga*	81%
Haemophilus	66%	*Kingella*	81%	*Kingella*	81%
Acidovorax	65%	*Magnetospirillum*	80%	*Ruegeria*	81%
Nitrosospira	61%	*Hyphomicrobium*	79%	*Chryseobacterium*	79%
Nitrosomonas	60%	*Campylobacter*	78%	*Campylobacter*	78%
Brevibacillus	60%	*Flavobacterium*	77%	*Flavobacterium*	78%
Shewanella	59%	*Leptospira*	77%	*Halorubrum*	78%
Actinomyces	58%	*Prevotella*	77%	*Myroides*	78%
Methylobacterium	58%	*Sulfurimonas*	73%	*Shewanella*	78%
Nitrobacter	52%	*Azospirillum*	71%	*Sulfurimonas*	78%
Afipia	50%	*Paracoccus*	71%	*Leptospira*	77%
Azospirillum	49%	*Sinorhizobium*	70%	*Paracoccus*	76%
Geobacillus	48%	*Thalassospira*	70%	*Roseobacter*	75%
Bradyrhizobium	46%	*Achromobacter*	69%	*Sinorhizobium*	72%
Hyphomicrobium	44%	*Bacillus*	65%	*Achromobacter*	71%
Bacillus	43%	*Rhizobiales*	60%	*Thalassospira*	71%
		Thauera	56%	*Haloferax*	65%
				Bacillus	63%
				Thauera	59%

The nucleotide-sequence fragments between the PCR primers used for pyrosequencing were analyzed. For each gene, the median value (in bold) among all of the genera analyzed was used as the cutoff value to cluster sequences into OTUs.

Supplementary Table S6. Summary of the alpha-diversities of bacterial communities in soil samples

Genes	*Incubation time*	*Soil depths*	*Soil core*	*No. of filtered sequences*	*No. of sequences for analysis*	*Good's library coverage*	*No. of OTUs observed (OTU richness)*	*No. of OTUs estimated (Chao1 richness)**	*Inverse Simpson index**	*Shannon's diversity index (H')**	*Shannon's species evenness (E)**
	-99 h	0–1 cm	1	2149	1841	98.0%	157	178 (167, 206)	27.6 (25.7, 29.7)	3.9 (3.9, 4)	0.78 (0.76, 0.79)
	-99 h	0–1 cm	2	1841	1841	97.1%	162	210 (186, 257)	25.7 (23.7, 28.1)	3.9 (3.8, 3.9)	0.76 (0.75, 0.78)
	-99 h	0–1 cm	3	2098	1841	98.0%	127	156 (140, 192)	22 (20.4, 23.9)	3.7 (3.6, 3.7)	0.76 (0.74, 0.77)
	-99 h	1–3 cm	1	2137	1841	96.8%	162	219 (192, 272)	22.1 (20, 24.6)	3.8 (3.8, 3.9)	0.76 (0.74, 0.77)
	-99 h	1–3 cm	2	2155	1841	97.7%	132	173 (151, 220)	9.8 (9, 10.7)	3.2 (3.1, 3.3)	0.65 (0.64, 0.67)
	-99 h	1–3 cm	3	2031	1841	98.2%	108	134 (119, 167)	11.6 (10.8, 12.6)	3.2 (3.1, 3.2)	0.67 (0.66, 0.69)
	-99 h	3–5 cm	1	2235	1841	97.3%	140	189 (164, 240)	8.2 (7.4, 9.1)	3.2 (3.1, 3.3)	0.64 (0.63, 0.66)
	-99 h	3–5 cm	2	2212	1841	96.8%	135	227 (181, 319)	8.7 (7.9, 9.6)	3.1 (3.1, 3.2)	0.64 (0.62, 0.65)
nirK in cluster II	-99 h	3–5 cm	3	2444	1841	97.6%	129	167 (147, 209)	9.4 (8.6, 10.4)	3.1 (3.1, 3.2)	0.65 (0.63, 0.66)
	24 h	0–1 cm	1	2336	1841	98.1%	131	157 (142, 190)	19.6 (18.1, 21.4)	3.7 (3.6, 3.7)	0.75 (0.74, 0.76)
	24 h	0–1 cm	2	2506	1841	97.9%	108	149 (126, 201)	5.4 (5, 5.9)	2.8 (2.7, 2.9)	0.59 (0.57, 0.61)
	24 h	0–1 cm	3	2918	1841	97.6%	124	171 (146, 225)	8.7 (8, 9.7)	3.1 (3, 3.2)	0.64 (0.63, 0.66)
	24 h	1–3 cm	1	2747	1841	97.2%	137	188 (162, 240)	18 (16.8, 19.4)	3.5 (3.4, 3.6)	0.71 (0.7, 0.73)
	24 h	1–3 cm	2	2559	1841	97.2%	143	194 (168, 245)	14.9 (13.7, 16.2)	3.5 (3.4, 3.5)	0.7 (0.68, 0.71)
	24 h	1–3 cm	3	2289	1841	98.0%	107	133 (119, 167)	6.9 (6.3, 7.6)	2.9 (2.8, 3)	0.62 (0.6, 0.64)
	24 h	3–5 cm	1	2440	1841	96.8%	154	213 (184, 269)	11.1 (10.1, 12.4)	3.4 (3.4, 3.5)	0.68 (0.67, 0.7)
	24 h	3–5 cm	2	2304	1841	97.1%	142	207 (174, 272)	11.9 (11, 13)	3.3 (3.2, 3.4)	0.67 (0.65, 0.68)
	24 h	3–5 cm	3	2822	1841	97.6%	124	161 (141, 201)	10.7 (10, 11.6)	3.1 (3, 3.2)	0.64 (0.63, 0.66)

	-99 h	0–1 cm	1	6757	5855	99.9%	66	70 (67, 84)	5.7 (5.5, 6)	2.5 (2.5, 2.6)	0.61 (0.6, 0.62)
	-99 h	0–1 cm	2	6206	5855	99.9%	59	62 (60, 75)	4.4 (4.2, 4.5)	2.1 (2.1, 2.2)	0.52 (0.51, 0.53)
	-99 h	0–1 cm	3	6589	5855	99.9%	62	67 (63, 85)	11.1 (10.8, 11.5)	2.8 (2.8, 2.8)	0.68 (0.67, 0.69)
	-99 h	1–3 cm	1	6735	5855	99.7%	68	106 (80, 194)	6.6 (6.4, 6.8)	2.4 (2.4, 2.5)	0.58 (0.57, 0.59)
	-99 h	1–3 cm	2	6825	5855	99.8%	57	70 (60, 108)	6.3 (6.1, 6.6)	2.4 (2.3, 2.4)	0.58 (0.58, 0.59)
	-99 h	1–3 cm	3	7027	5855	99.9%	43	57 (46, 110)	4.6 (4.5, 4.8)	2 (2, 2)	0.54 (0.53, 0.54)
	-99 h	3–5 cm	1	6385	5855	99.8%	75	82 (77, 100)	11.8 (11.4, 12.2)	2.9 (2.9, 3)	0.68 (0.67, 0.68)
	-99 h	3–5 cm	2	5855	5855	99.9%	72	75 (73, 86)	9.8 (9.4, 10.1)	2.8 (2.8, 2.8)	0.65 (0.64, 0.66)
nirS in cluster I	-99 h	3–5 cm	3	6246	5855	99.7%	63	84 (69, 136)	7.2 (6.9, 7.5)	2.5 (2.5, 2.5)	0.61 (0.6, 0.61)
	24 h	0–1 cm	1	6056	5855	99.9%	65	68 (66, 81)	5.5 (5.3, 5.8)	2.4 (2.4, 2.5)	0.59 (0.58, 0.59)
	24 h	0–1 cm	2	6686	5855	99.7%	63	78 (67, 114)	6.2 (6, 6.3)	2.3 (2.2, 2.3)	0.54 (0.54, 0.55)
	24 h	0–1 cm	3	6708	5855	99.8%	52	59 (54, 84)	4 (3.9, 4.2)	2 (2, 2.1)	0.52 (0.51, 0.53)
	24 h	1–3 cm	1	6164	5855	99.8%	57	66 (59, 93)	8.2 (7.9, 8.4)	2.5 (2.4, 2.5)	0.61 (0.61, 0.62)
	24 h	1–3 cm	2	7086	5855	99.8%	66	72 (67, 90)	7.8 (7.5, 8.1)	2.5 (2.5, 2.6)	0.61 (0.6, 0.62)
	24 h	1–3 cm	3	6318	5855	99.9%	39	39 (39, 43)	4.5 (4.4, 4.6)	1.9 (1.8, 1.9)	0.51 (0.5, 0.51)
	24 h	3–5 cm	1	6685	5855	99.8%	74	92 (79, 139)	8.9 (8.6, 9.3)	2.8 (2.7, 2.8)	0.64 (0.63, 0.65)
	24 h	3–5 cm	2	6482	5855	99.8%	77	85 (79, 104)	8.7 (8.4, 9.1)	2.7 (2.6, 2.7)	0.62 (0.61, 0.63)
	24 h	3–5 cm	3	6460	5855	99.8%	61	68 (63, 86)	6 (5.8, 6.2)	2.3 (2.3, 2.4)	0.57 (0.56, 0.57)

	-99 h	0–1 cm	1	6281	6281	99.8%	98	103 (99, 120)	19.7 (19.1, 20.3)	3.4 (3.3, 3.4)	0.74 (0.73, 0.74)
	-99 h	0–1 cm	2	8326	6281	99.7%	105	122 (110, 158)	15.7 (15.1, 16.4)	3.3 (3.2, 3.3)	0.7 (0.69, 0.71)
	-99 h	0–1 cm	3	7729	6281	99.7%	85	98 (89, 129)	12.6 (12.2, 13.1)	3 (3, 3)	0.68 (0.67, 0.68)
	-99 h	1–3 cm	1	7804	6281	99.7%	103	112 (106, 133)	17.4 (16.8, 18)	3.3 (3.2, 3.3)	0.71 (0.7, 0.71)
	-99 h	1–3 cm	2	7311	6281	99.8%	92	113 (98, 165)	11.9 (11.5, 12.3)	3 (3, 3)	0.67 (0.66, 0.67)
	-99 h	1–3 cm	3	9137	6281	99.7%	97	118 (104, 162)	11.1 (10.6, 11.6)	3.1 (3, 3.1)	0.67 (0.66, 0.68)
	-99 h	3–5 cm	1	7464	6281	99.6%	100	132 (111, 189)	10.9 (10.5, 11.3)	3 (2.9, 3)	0.64 (0.63, 0.65)
	-99 h	3–5 cm	2	6943	6281	99.8%	99	107 (101, 127)	12.9 (12.5, 13.3)	3 (3, 3.1)	0.66 (0.65, 0.67)
nosZ in clade I	-99 h	3–5 cm	3	8367	6281	99.8%	92	100 (94, 118)	12.5 (12.1, 12.9)	3 (3, 3.1)	0.67 (0.66, 0.67)
	24 h	0–1 cm	1	8058	6281	99.8%	89	95 (90, 113)	8.1 (7.7, 8.5)	2.9 (2.9, 2.9)	0.65 (0.64, 0.66)
	24 h	0–1 cm	2	8402	6281	99.7%	89	104 (94, 137)	11 (10.6, 11.4)	3 (2.9, 3)	0.66 (0.66, 0.67)
	24 h	0–1 cm	3	7484	6281	99.9%	84	89 (85, 105)	13.1 (12.6, 13.6)	3.1 (3.1, 3.1)	0.7 (0.69, 0.71)
	24 h	1–3 cm	1	10360	6281	99.8%	91	99 (93, 117)	11.6 (11.2, 12.1)	3 (2.9, 3)	0.66 (0.65, 0.67)
	24 h	1–3 cm	2	8968	6281	99.7%	99	116 (104, 152)	12.4 (12, 12.9)	3 (3, 3.1)	0.66 (0.66, 0.67)
	24 h	1–3 cm	3	7798	6281	99.9%	70	71 (70, 79)	3.8 (3.7, 3.9)	2 (1.9, 2)	0.46 (0.45, 0.47)
	24 h	3–5 cm	1	8558	6281	99.7%	107	128 (114, 169)	13.7 (13.2, 14.2)	3.1 (3.1, 3.2)	0.67 (0.66, 0.68)
	24 h	3–5 cm	2	8872	6281	99.7%	102	118 (107, 149)	13.8 (13.4, 14.3)	3.1 (3.1, 3.1)	0.67 (0.66, 0.67)
	24 h	3–5 cm	3	8789	6281	99.7%	97	118 (104, 159)	9.4 (9, 9.9)	2.9 (2.9, 2.9)	0.63 (0.62, 0.64)

	-99 h	0–1 cm	1	3119	1826	96.8%	159	240 (200, 319)	25.5 (23.4, 27.9)	3.9 (3.8, 4)	0.77 (0.76, 0.78)
	-99 h	0–1 cm	2	2387	1826	95.6%	212	302 (263, 372)	37.1 (34.2, 40.7)	4.3 (4.2, 4.3)	0.8 (0.79, 0.81)
	-99 h	0–1 cm	3	2815	1826	96.7%	155	251 (204, 345)	17.5 (16.1, 19.2)	3.7 (3.6, 3.7)	0.73 (0.71, 0.74)
	-99 h	1–3 cm	1	2395	1826	95.3%	207	321 (272, 408)	27.5 (25.2, 30.3)	4.1 (4, 4.2)	0.77 (0.75, 0.78)
	-99 h	1–3 cm	2	2508	1826	95.6%	188	313 (257, 413)	20.2 (18.3, 22.6)	3.9 (3.8, 3.9)	0.74 (0.72, 0.75)
	-99 h	1–3 cm	3	1902	1826	96.5%	163	241 (204, 310)	17.9 (16.3, 19.8)	3.7 (3.7, 3.8)	0.73 (0.72, 0.75)
	-99 h	3–5 cm	1	2333	1826	95.4%	191	336 (271, 453)	29.1 (27, 31.6)	4 (3.9, 4.1)	0.76 (0.75, 0.78)
	-99 h	3–5 cm	2	2245	1826	94.6%	206	373 (303, 494)	28.6 (26.4, 31.2)	4 (4, 4.1)	0.76 (0.74, 0.77)
nosZ in clade II	-99 h	3–5 cm	3	2358	1826	96.2%	180	261 (224, 329)	25.6 (23.6, 27.9)	3.9 (3.8, 4)	0.75 (0.74, 0.77)
	24 h	0–1 cm	1	3133	1826	97.0%	157	231 (194, 306)	25.8 (23.8, 28.1)	3.9 (3.8, 3.9)	0.77 (0.75, 0.78)
	24 h	0–1 cm	2	2877	1826	97.0%	136	229 (181, 326)	16 (14.8, 17.4)	3.5 (3.4, 3.5)	0.7 (0.69, 0.72)
	24 h	0–1 cm	3	2376	1826	96.8%	148	220 (185, 289)	18.8 (17.5, 20.2)	3.6 (3.5, 3.7)	0.72 (0.7, 0.73)
	24 h	1–3 cm	1	2266	1826	96.3%	169	245 (210, 310)	13 (11.8, 14.4)	3.5 (3.5, 3.6)	0.69 (0.68, 0.71)
	24 h	1–3 cm	2	2276	1826	95.7%	163	420 (292, 674)	17 (15.6, 18.8)	3.6 (3.6, 3.7)	0.72 (0.7, 0.73)
	24 h	1–3 cm	3	1826	1826	97.4%	128	191 (158, 259)	13.3 (12.4, 14.4)	3.3 (3.2, 3.4)	0.68 (0.67, 0.7)
	24 h	3–5 cm	1	2449	1826	96.0%	185	267 (230, 335)	23.6 (21.8, 25.8)	3.9 (3.8, 4)	0.75 (0.73, 0.76)
	24 h	3–5 cm	2	2620	1826	96.8%	165	222 (195, 275)	20.9 (19.4, 22.7)	3.8 (3.7, 3.8)	0.74 (0.72, 0.75)
	24 h	3–5 cm	3	2288	1826	96.1%	169	282 (229, 381)	16.2 (15, 17.7)	3.6 (3.5, 3.7)	0.71 (0.69, 0.72)

Abbreviations: No., number; OTU, operational taxonomic unit.

Asterisks (*) indicate that the data are presented as means with upper and lower 95% confidence intervals in parentheses.

Supplementary Table S7. The *p*-values in the statistical analysis for alpha-diversity indices of *nirK* in cluster II

			p-value		
Incubation time	*Soil depths*	*Statistical analysis*	*Inverse Simpson index*	*Shannon's diversity index (H')*	*Shannon's species evenness (E)*
−99 h	0–5 cm	One-way ANOVA	**0.008**	**0.019**	**0.013**
	0–1 cm vs. 1–3 cm	Tukey's post-test	**0.048**	0.104	0.091
	0–1 cm vs. 3–5 cm	Tukey's post-test	**0.007**	**0.016**	**0.011**
	1–3 cm vs. 3–5 cm	Tukey's post-test	0.288	0.343	0.258

Items with a $p < 0.05$ are in bold.

Supplementary Table S8. Physicochemical properties of the soil samples

Sampling time	*Depth (Distance from the surface)*	*Water-soluble carbon (mg C kg^{-1} soil)*	*Nitrogen (Nitrate-N mg kg^{-1} soil)*	*pH*	*Core ID*
−99 h	0-1 cm	460	18.1	5.33	1
−99 h	0-1 cm	320	34.9	4.94	2
−99 h	0-1 cm	310	92.1	4.86	3
−99 h	1-3 cm	210	11.2	5.01	1
−99 h	1-3 cm	180	31.5	4.77	2
−99 h	1-3 cm	200	14.1	4.84	3
−99 h	3-5 cm	190	6.5	5.03	1
−99 h	3-5 cm	170	13.8	4.89	2
−99 h	3-5 cm	200	8.9	4.88	3

Supplementary Table S9. Correlation between the alpha-diversity indices of the denitrifiers harboring *nirK* in cluster II and environmental factors

Environmental factors	*Inverse Simpson index*		*Shannon's diversity index (H')*		*Shannon's species evenness (E)*	
	ρ	*p-value*	*ρ*	*p-value*	*ρ*	*p-value*
Vertical distance (soil depth)	**-0.896**	**0.001**	**-0.843**	**0.004**	**-0.949**	**< 0.001**
Horizontal distance (soil core)	-0.158	0.685	-0.474	0.197	-0.105	0.787
Water-soluble carbon	**0.887**	**0.001**	**0.828**	**0.006**	**0.937**	**< 0.001**
Nitrate-N	0.600	0.097	0.500	0.178	0.650	0.067
pH	0.250	0.521	0.450	0.230	0.233	0.552

Abbreviations: *ρ*, Spearman's rho (Spearman's rank correlation coefficient).

Items with a $p < 0.05$ are in bold.

Supplementary Table S10. Summary of PERMANOVA tests assessing relationships between the beta-diversities of bacteria and environmental factors

Genes	*Environmental factors*	*PERMANOVA test*		
		F value	R^2	*p-value*
nirK in cluster II	Soil depth	8.49	0.55	**0.002**
	Soil core	0.67	0.09	0.577
	Water-soluble carbon	5.12	0.42	**0.011**
	Nitrogen (Nitrate-N)	3.66	0.34	**0.026**
	pH	1.35	0.16	0.256
nirS in cluster I	Soil depth	6.29	0.47	**0.003**
	Soil core	0.78	0.10	0.558
	Water-soluble carbon	5.40	0.44	**0.007**
	Nitrogen (Nitrate-N)	4.47	0.39	**0.032**
	pH	1.60	0.19	0.216
nosZ in clade I	Soil depth	7.60	0.52	**0.002**
	Soil core	0.94	0.12	0.425
	Water-soluble carbon	5.13	0.42	**0.022**
	Nitrogen (Nitrate-N)	3.89	0.36	**0.017**
	pH	1.55	0.18	0.219
nosZ in clade II	Soil depth	7.07	0.50	**0.004**
	Soil core	0.91	0.12	0.437
	Water-soluble carbon	5.59	0.44	**0.003**
	Nitrogen (Nitrate-N)	2.61	0.27	**0.042**
	pH	1.59	0.18	0.179

Items with a $p < 0.05$ are in bold.

Supplementary Table S11. Dominant OTUs enriched at different soil depths

Genes	*Soil depths*	*OTUs*	*Taxonomy*
nirK in cluster II	0-1 cm	OTU6	*Chthoniobacter*
		OTU10	*Ralstonia*
	3-5 cm	OTU1	*Salinisphaera*
		OTU5	*Methylocella*
		OTU14	*Hyphomicrobium*
nirS in cluster I	0-1 cm	OTU12	*Thiothrix*
	1-3 cm	OTU2	*Bradyrhizobium*
	3-5 cm	OTU7	*Cupriavidus*
		OTU11	*Bradyrhizobium*
		OTU15	*Competibacter*
nosZ in clade I	0-1 cm	OTU14	*Azospirillum*
		OTU10	*Azospirillum*
		OTU15	*Bradyrhizobium*
		OTU6	*Bradyrhizobium*
	3-5 cm	OTU2	*Bradyrhizobium*
		OTU5	*Bradyrhizobium*
		OTU16	*Microvirga*
		OTU19	*Mesorhizobium*
nosZ in clade II	0-1 cm	OTU3	*Flavobacterium*
		OTU6	*Flavihumibacter*
		OTU12	*Melioribacter*
	1-3 cm	OTU1	*Melioribacter*
	3-5 cm	OTU4	*Ignavibacterium*
		OTU5	*Ardenticatena*
		OTU8	*Opitutus*
		OTU19	*Anaeromyxobacter*

Supplementary Table S12. Summary of weighted UniFrac analyses

Genes	*Soil depth*	*UniFrac distance* (–99 h vs. 24 h)	*p-value*
	0-1 cm	0.938	**<0.001**
nirK-CII	1-3 cm	0.659	**<0.001**
	3-5 cm	0.802	**<0.001**
	0-1 cm	0.647	**<0.001**
nirS-CI	1-3 cm	0.634	**<0.001**
	3-5 cm	0.797	0.053
	0-1 cm	0.834	**<0.001**
nosZ-CI	1-3 cm	0.635	**<0.001**
	3-5 cm	0.773	0.116
	0-1 cm	0.798	**<0.001**
nosZ-CII	1-3 cm	0.630	**<0.001**
	3-5 cm	0.747	**<0.001**

Items with a $p < 0.05$ are in bold.

Supplementary Table S13. OTUs exhibiting significantly altered abundance in soils after waterlogging

Genes	*Soil depth*	*OTUs*	*Fold change of abundance**	*p-value*	*Taxonomy*
nirK in cluster II	0-1cm	OTU6	2.8	0.0495	*Chthoniobacter*
	1-3cm	OTU5	2.2	0.0495	*Methylocella*
		OTU16	5.4	0.0495	*Neisseria*
		OTU3	−1.7	0.0495	*Methylocella*
		OTU9	−1.7	0.0495	*Sphaerobacter*
		OTU15	−2.4	0.0495	*Sphaerobacter*
	3-5cm	OTU2	5.3	0.0495	*Caulobacter*
		OTU13	2.4	0.0463	*Mesorhizobium*
		OTU17	2.8	0.0495	*Rhodanobacter*
		OTU1	−1.3	0.0495	*Salinisphaera*
		OTU7	−2.0	0.0495	*Sphaerobacter*
		OTU15	−3.0	0.0495	*Sphaerobacter*
nirS in cluster I	3-5cm	OTU4	14.9	0.0495	*Pelomonas*
		OTU6	1.4	0.0495	*Competibacter*
		OTU12	3.9	0.0463	*Thiothrix*
		OTU7	−1.7	0.0495	*Cupriavidus*
		OTU11	−2.6	0.0495	*Bradyrhizobium*
nosZ in clade I	0-1cm	OTU6	2.4	0.0495	*Bradyrhizobium*
		OTU11	−2.2	0.0495	*Bradyrhizobium*
	1-3cm	OTU15	−6.1	0.0495	*Bradyrhizobium*
	3-5cm	OTU4	5.8	0.0495	*Albidiferax*
		OTU11	1.5	0.0463	*Bradyrhizobium*
		OTU19	−1.2	0.0495	*Mesorhizobium*

nosZ in clade II	0-1cm	OTU1	3.2	0.0495	*Melioribacter*
		OTU3	1.4	0.0495	*Flavobacterium*
		OTU5	−7.8	0.0495	*Ardenticatena*
		OTU19	−2.6	0.0463	*Anaeromyxobacter*
	1-3cm	OTU12	−6.8	0.0495	*Melioribacter*
		OTU13	−6.0	0.0495	*Bacillus*
	3-5cm	OTU1	1.4	0.0495	*Melioribacter*
		OTU2	3.1	0.0495	*Ignavibacterium*
		OTU5	−2.7	0.0495	*Ardenticatena*
		OTU17	−1.7	0.0495	*Opitutus*

* Negative values indicate a decrease in abundance.
